# 1,25(OH)_2_D_3_ regulates the proangiogenic activity of pericyte through VDR‐mediated modulation of VEGF production and signaling of VEGF and PDGF receptors

**DOI:** 10.1096/fba.2018-00067

**Published:** 2019-05-21

**Authors:** Nasim Jamali, Yong‐Seok Song, Christine M. Sorenson, Nader Sheibani

**Affiliations:** ^1^ Department of Ophthalmology and Visual Sciences University of Wisconsin School of Medicine and Public Health Madison Wisconsin; ^2^ McPherson Eye Research Institute, University of Wisconsin School of Medicine and Public Health Madison Wisconsin; ^3^ Department of Pediatrics University of Wisconsin School of Medicine and Public Health Madison Wisconsin; ^4^ Department of Cell and Regenerative Biology University of Wisconsin School of Medicine and Public Health Madison Wisconsin; ^5^ Department of Biomedical Engineering University of Wisconsin School of Medicine and Public Health Madison Wisconsin

**Keywords:** angiogenesis, cell adhesion, pericytes, retinal vasculature, signal transduction, vitamin D

## Abstract

We have previously demonstrated that the active form of vitamin D (calcitriol; 1,25(OH)_2_D_3_) is a potent inhibitor of retinal neovascularization. However, the underlying molecular and cellular mechanisms involved remained poorly understood. Perivascular supporting cells including pericytes (PC) play important roles during angiogenesis, vascular maturation, and stabilization of blood vessels. How 1,25(OH)_2_D_3_ affects retinal PC proliferation and migration, and whether these effects are mediated through vitamin D receptor (VDR), are unknown. Here, we determined the impact of 1,25(OH)_2_D_3_ on retinal PC prepared from wild‐type (Vdr+/+) and VDR‐deficient (Vdr−/−) mice. Retinal PC expressed significantly higher VDR levels compared to retinal endothelial cells (EC). Unlike retinal EC, 1,25(OH)_2_D_3_ significantly decreased PC proliferation and migration and resulted in a G_0_/G_1_ cell cycle arrest. Although 1,25(OH)_2_D_3_ did not inhibit the proliferation of Vdr−/− PC, it did inhibit their migration. PC adhesion to various extracellular matrix (ECM) proteins and ECM production were also affected by incubation of PC with 1,25(OH)_2_D_3_. Vdr−/− PC were more adherent compared with Vdr+/+ cells. Mechanistically, incubation of Vdr+/+ PC with 1,25(OH)_2_D_3_ resulted in an increased expression of vascular endothelial growth factor (VEGF) and attenuation of signaling through VEGF‐R2 and platelet‐derived growth factor receptor‐beta. Incubation with soluble VEGF‐R1 (sFlt‐1) partially reversed the effect of VEGF on Vdr+/+ PC. In addition, incubation of Vdr+/+ PC with VEGF or inhibition of VEGF‐R2 increased VDR expression. Together, these results suggest an important role for retinal PC as a target for vitamin D and VDR action for attenuation of angiogenesis.

**Abbreviations**
AKT/PKB: protein kinase B
CM: conditioned medium
EC: endothelial cells
EDTA: ethylenediaminetetraacetic acid
ELISA: enzyme-linked immunosorbent assay
ERK: extracellular-signal-regulated kinases
FACS: flow activated cell sorting
FITC: fluorescein isothiocyanate
HEPES: 4-(2-hydroxyethyl)-1-piperazineethanesulfonic acid VDR: Vitamin D receptor
IFN-γ: interferon-gama
JNK: jun-N-terminal kinase
MAPK: mitogen activated protein kinases
OIR: oxygen-induced ischemic retinopathy
PC: pericytes
PDGF: platelet-derived growth factor
PDFG-Rβ: PDGF receptor beta
qPCR: quantitative polymerase chain reaction
SDS-PAGE: sodium dodecyl sulfate-polyacrylamide gel electrophoresis
SMA: smooth muscle actin
sFLT-1: soluble FLT-1
VDRE: vitamin D response element
VSMC: vascular smooth muscle cells
VEGF: vascular endothelial growth factor
VEGF-R2: VEGF receptor-2/FLK-1
VEGF-R1: VEGF receptor-1/FLT-1

## 1 INTRODUCTION

Angiogenesis, the formation of new blood vessels from preexisting capillaries, occurs during development, reproduction, and tissue regeneration and repair processes. A tightly balanced production of positive and negative regulatory factors normally keep angiogenesis in check. However, this regulation becomes abrogated under pathological conditions such as cancer, diabetes, age‐related macular degeneration, and autoimmune diseases leading to neovascularization.^1^ In blood vessels, endothelial cells (EC) line the inside of the lumen and perivascular supporting cells, including smooth muscle cells and pericytes (SMC/PC) embed themselves in a shared basement membrane with EC. SMC/PC are known to mediate vascular stability and control EC proliferation and survival. They are also involved in vascular development, stabilization, and remodeling.^2^ A PC to EC ratio of 1:1 in the human retinal vasculature is greater than in any other organ.^3^ Thus, it is not surprising that alterations in PC function contribute to several pathologies of the central nervous system including diabetic retinopathy, multiple sclerosis, stroke, and Alzheimer's disease.^4^

In the newly forming blood vessels, EC secrete platelet‐derived growth factor (PDGF) recruiting PDGF‐Rβ–positive PC to nascent sprouts. PC in return, secrete vascular endothelial growth factor (VEGF) to promote EC survival.^5^ Previous studies indicated that 1,25(OH)_2_D_3_ increases VEGF production in vascular SMC.^5^, ^6^ However, the consequence of this increased VEGF level on PC function remained unclear. In addition, how quiescent characteristic of PC is established following their recruitment to newly formed vessels remained unknown. Cheresh and colleagues showed incubation of PC with VEGF has a negative effect on their proliferation and migration in response to PDGF.^7^ This effect was demonstrated to be mediated through enhanced VEGF‐R2 heterodimerization with, and inactivation of, PDGF‐Rβ on PC, attenuating PC proangiogenic activity and promoting blood vessel maturity. Thus, the production of VEGF by PC, acting in an autocrine fashion, signals the quiescence of PC on developing vessels.

We previously demonstrated that 1,25(OH)_2_D_3_ is a potent inhibitor of retinal neovascularization during oxygen‐induced ischemic retinopathy (OIR).^8^ Furthermore, we recently showed this inhibition of angiogenesis is vitamin D receptor (VDR) dependent.^9^ VDR is a member of the nuclear receptor superfamily of ligand‐activated transcription factors. Upon its activation by 1,25(OH)_2_D_3_, it forms a heterodimer with one of the family of retinoid X receptors (RXRs), and perhaps other co‐regulatory factors, which impact its expression and binding to the vitamin D response element (VDRE) in its target gene.^10^ PC play a fundamental role in EC survival and stabilization of newly forming blood vessels by facilitating VEGF production, migration, and guiding a path for growing sprouts.^11^, ^12^ Pericyte recruitment and migration are necessary for angiogenesis, and the inhibition of PC migration is crucial in suppressing angiogenesis. How 1,25(OH)_2_D_3_ inhibits angiogenesis, and more specifically, affects retinal vascular cell function, including EC and PC, remained largely unknown.

We previously showed that 1,25(OH)_2_D_3_ minimally affects proliferation and migration of retinal EC in culture,^8^ consistent with the lack of vitamin D–mediated osteoblastic differentiation in EC.^13^ Here, we tested the hypothesis that 1,25(OH)_2_D_3_ inhibits PC proliferation and migration through increased VEGF production, thus attenuating the proangiogenic activity of PC. We showed that both retinal EC and PC express VDR, with PC expressing significantly higher levels. We also showed that 1,25(OH)_2_D_3_ inhibited retinal PC proliferation and migration, and increased PC adhesion. These changes were concomitant with the cell cycle arrest in PC incubated with 1,25(OH)_2_D_3_. However, 1,25(OH)_2_D_3_ failed to inhibit the Vdr−/− PC proliferation. Here, we demonstrate that VEGF level is significantly increased in PC incubated with 1,25(OH)_2_D_3_. In addition, the addition of exogenous VEGF increased VDR levels, and attenuated PDGF‐mediated PC migration. Furthermore, the addition of the VEGF trap (sFLT‐1; VEGF antagonist) restored the basal VDR expression and migration of PC incubated with 1,25(OH)_2_D_3_. Thus, the presence of VEGF negatively impacts PDGF‐mediated PC proliferation and migration, likely through inactivation of signaling through VEGF and PDGF receptors. In support of this observation, we showed a significant increase in colocalization/proximity of VEGF‐R2 and PDGF‐Rβ in PC incubated with 1,25(OH)_2_D_3_. Thus, 1,25(OH)_2_D_3_‐mediated VEGF production in PC promotes VEGF‐R2 and PDGF‐Rβ interactions, attenuation of signaling through these receptors, and a quiescence and mature vascular phenotype.

## 2 MATERIALS AND METHODS

### 2.1 Ethics statement

All animal experiments were performed in accordance to the Association for Research in Vision and Ophthalmology Statement for the Use of Animals in Ophthalmic and Vision Research and were approved by the Institutional Animal Care and Use Committee of the University of Wisconsin School of Medicine and Public Health (the assurance number A3368‐01). Animals were sacrificed according to an approved protocol by CO_2_ asphyxiation.

### 2.2 Experimental animals, tissue preparation, and culture of retinal PC

Immortomice expressing a temperature‐sensitive SV40 large T antigen were purchased from Charles River Laboratories (Wilmington, MA) and backcrossed to C57BL/6J mice for 10 generations. The VDR‐deficient (Vdr−/−) mice (B6.129S4‐Vdr^tm1Mbd^/J; Jackson Laboratories, Bar Harbor, ME; stock number 006133) were crossed with the Immortomice and screened as described previously.^14^ To isolate PC, retinas were dissected from one litter of Vdr−/− Immortomice (6‐7 pups, 4 weeks old) using a dissecting microscope. Retinas were then rinsed with serum‐free Dulbecco's Modified Eagle Medium (DMEM; Invitrogen, Carlsbad, CA), pooled in a 60‐mm dish, minced, and digested for 45 minutes with collagenase type II (1 mg/mL; Worthington, Lakewood, NJ) with 0.1% bovine serum albumin (BSA) in serum‐free DMEM at 37°C. Cells were rinsed in DMEM containing 10% fetal bovine serum (FBS) and centrifuged for 5 minutes at 400×g. The digested tissue was resuspended in 4 mL DMEM containing 10% FBS, 2 mmol/L l‐glutamine, 100 μg/mL streptomycin, 100 U/mL penicillin, and murine recombinant IFN‐*γ* (R&D Systems, Minneapolis, MN) at 44 U/mL. Cells were then divided into four wells of a 24‐well tissue culture plate evenly and maintained at 33°C with 5% CO_2_. Cells were then gradually passed to larger plates, maintained, and propagated in 60‐mm tissue culture dishes. These cells express a temperature‐sensitive large T antigen whose expression is induced in the presence of interferon‐gama (IFN‐γ) allowing the cells to readily propagate when cultured at 33°C. The culture of these cells at 37°C in the absence of IFN‐γ for 48 hours results in loss of large T antigen. Here, all the experiments were conducted with at least two different isolation of retinal PC and repeated at least once (N ≥ 4).

### 2.3 FACS analysis

Flow cytometry analysis was used to assess the expression of PC makers, cell cycle, VEGF receptors, colocalization of VEGF‐R2 and PDGF‐Rβ, and expression of integrins in PC. Confluent 60‐mm culture plates of cells were rinsed with phosphate‐buffered saline (PBS) containing 0.04% Ethylenediaminetetraacetic acid (EDTA) and incubated with 1.5 mL of cell dissociation solution (tris‐buffered saline [TBS; 20 mmol/L Tris‐HCl and 150 mmol/L NaCl; pH 7.6] containing 2 mmol/L EDTA and 0.05% BSA). Cells were then collected from plates with DMEM containing 10% FBS centrifuged and washed once with 5 mL of TBS, and blocked in 0.5 mL of TBS with 1% goat serum for 20 minutes on ice. Cells were centrifuged for 5 minutes at 400×g and resuspended and incubated in 0.5 mL TBS with 1% BSA containing appropriate dilution of primary antibody (as recommended by supplier), and incubated on ice for 30 minutes. The following antibodies were used: rabbit anti‐NG2 (Cat#: AB5320; Millipore, Temecula, CA), rabbit anti‐mouse α‐smooth muscle actin (Cat#: F3777; Sigma‐Aldrich, St Louis, MO), rat anti‐mouse CD140b/PDGF‐Rβ (Cat#: 14‐1402; eBiosciences), rabbit anti‐mouse anti‐PDGF‐Rβ (Cat#: 3169; Cell Signaling), rat anti‐mouse anti‐PDGF‐Rβ (Cat#: LS‐C 107026/102757; Lifespan Biosciences), rat anti‐mouse anti‐VEGF‐R1/FLT‐1 (Cat#: MAB471; R&D Systems), rat anti‐mouse anti‐VEGF‐R2/FLK‐1 (Cat#: MAB4432; R&D Systems), rabbit anti‐mouse anti‐VEGF‐R2 (Clone D5B1, Cat#: 12687, AlexaFluor^®^ 488 conjugated; Cell signaling), VEGF‐R2/FLK‐1 (Cat#: PA1‐21025; Thermo Fisher, Rockford, IL), anti‐α3 (Cat#: sc‐6588, N‐19; Santa Cruz), anti‐α3 (Cat#: AB1920; Millipore), anti‐α2 (Cat#: AB1944; Chemicon), anti‐α2 (Cat#: sc‐9089, H‐293; Santa Cruz), anti‐α4 (Cat#: AB1924; Millipore), anti‐α4 (Cat#: sc‐14008, H‐210; Santa Cruz), anti‐β1 (Cat#: sc‐8978, M‐106; Santa Cruz), anti‐β5 (Cat#: sc‐5401, E‐19; Santa Cruz), anti‐β8 (Cat#: sc‐25714, H‐160; Santa Cruz), anti‐α5β1 (Cat#: MAB 1999; Millipore), and anti‐αvβ3 (Cat#: MAB 1976Z; Millipore).

Antibodies were used at dilutions recommended by the supplier. Cells were then rinsed twice with TBS containing 1% BSA and incubated with appropriate fluorescein isothiocyanate (FITC)‐conjugated secondary antibody (Pierce, Rockford, IL) prepared in TBS containing 1% BSA for 30 minutes on ice. Following incubation, cells were washed twice with TBS containing 1% BSA, resuspended in 0.5 mL of TBS with 1% BSA and analyzed by a flow activated cell sorting (FACS) can caliber flow cytometer (Becton Dickinson, Franklin Lakes, NJ), and analysis were performed by FlowJo (FLOWJO, LLC, Ashland, OR, versions 9 and 10). Colocalization experiments were performed using Amnis Image stream^X^ mk II^TM^ (Millipore) with acquisition software INSPIRE (V 200.1.388.0; EMD Millipore), and analysis was performed using IDEAS analysis software (version 6.2).

For cell cycle analysis, following incubation with cell dissociation solution, cells were washed twice with cold PBS. Cells were then resuspended in Krishan's reagent (0.05 mg/mL propidium iodide, 0.1% Na citrate, 0.02 mg/mL ribonuclease A, and 0.3% NP‐40; pH 8.3) at concentration of 2 × 10^6^/mL and vortexed, followed by 30 minutes to 1 hour incubation on ice and analyzed by a FACScan caliber flow cytometer (Becton Dickinson).

### 2.4 Cell proliferation

Cell proliferation was assessed in two ways: counting the number of cells every other days for 2 weeks and measuring the percentage of cells 5‐ethynyl‐2′‐deoxyuridine (EdU) positive undergoing DNA synthesis using a Click‐It‐EdU Alexa Flour 488 assay (Invitrogen). Retinal PC were plated at 1 × 10^4^ in 60‐mm tissue culture dishes in triplicates. Cell numbers were counted every other day in triplicates and fed on days they were not counted for the duration of experiment. The rate of DNA synthesis was also assessed using s Click‐IT EdU Alexa Fluor 488 kit, as recommended by the supplier (Invitrogen). The assay measures incorporated EdU, a nucleoside analog of thymidine, during cell proliferation. Cells (5 × 10^4^) were plated in 60‐mm tissue culture dishes and were incubated with 10 μmol/L EdU in PC medium for at least 1 hour at 33°C. Synthesized DNA was then analyzed by measuring incorporated EdU using the FACSscan Caliber flow cytometer (Becton Dickinson).

### 2.5 Apoptosis TUNEL assay

To determine the percentage of apoptotic cells, retinal PC were plated at 1 × 10^4^ cells per well of a four‐chamber slide (Cat#: PEZGS0416; Millipore). The next day, medium was replaced with medium containing 10 μmol/L 1,25(OH)_2_D_3_ or solvent ethanol‐treated, and cells were incubated for 48 hours. TdT‐dUTP terminal nick‐end labeling (TUNEL) staining was performed using Click‐IT TUNEL Alexa Flour 594, as recommended by the supplier (Cat#: C10246; Life Technologies). Positive cells were counted per five high power field (×200) using a fluorescence microscope and reported as a percentage of apoptotic cells relative to total number of cells per field. DNase I treatment was used as a positive control as recommended by the supplier.

### 2.6 Scratch wound assays

Retinal PC were plated in 60‐mm tissue culture dishes and allowed to reach confluence (2‐3 days). Plates were wounded using a 1‐mL micropipette tip. Cells then washed with DMEM medium containing 10% FBS twice to remove detached floating cells. Cells were fed with growth medium containing 1 μmol/L 5‐fluorouracil (Sigma) to minimize the contribution of cell proliferation in wound closure. Wound closure was monitored by phase microscopy every 24 hours until the wound is closed. Images were captured in digital format. Medium and treatments were refreshed after each imaging. The migrated distances as percentage of total distance were reported for quantitative assessment.

### 2.7 Transwell migration assays

Cell migration was assessed using a transwell migration assay. Briefly, the bottom side of 8‐μm pore size transwell inserts filters (6.5‐mm insert with 8.0 µm pore, Cat#: 3422, Corning) were coated with 2 µg/mL fibronectin (FN) in 1× PBS and incubated overnight at 4°C in a 24‐well plate. The bottom side of transwell inserts was rinsed with PBS the next morning, and were blocked with PBS containing 2% BSA (Cat#: 199898; MP Biomedicals, Solon, OH) for 1 hour at room temperature and then rinsed with PBS. Serum‐free DMEM and appropriate treatment concentration (0.5 mL total) was added to the bottom a 24‐well plate, and transwell insert filters were inserted into the appropriate wells. Cells (1 × 10^5^) were trypsinized and resuspended in 100 µL of serum‐free DMEM containing appropriate treatment, and were added to the top of each transwell membrane. Cells were allowed to pass through the filters for 4 hours in a 33°C tissue culture incubator. The medium was aspired from the 24‐well plate (bottom side of transwell inserts), and the upper side of membranes was wiped with a cotton swab. The migrated cells through the membrane were fixed with 4% paraformaldehyde, stained with hematoxylin and eosin, and then mounted on a slide. The number of migrated cells was counted for 10 high‐power fields (×200) for each condition, and the average and SD were determined for the means. All samples were prepared in duplicates.

### 2.8 Cell adhesion to various extracellular matrix proteins assays

Cell adhesion assays were assessed using 96‐well flat bottom plates (Nunc Immunoplate, Maxisorp; Fisher Scientific). Plate was coated with various concentration of matrix proteins. Collagen I (Col I), collagen IV (Col IV), vitronectin (VN), and FN (BD Biosciences) were serially diluted in TBS with 2 mmol/L CaCl_2_ and 2 mmol/L MgCl_2_ (TBS with Ca/Mg) and coated the 96‐well plate (50 μL/well; TBS with Ca/Mg) overnight at 4°C. Plates were washed four times (200 μL/well) by TBS with Ca/Mg, blocked using TBS with Ca/Mg containing 1% BSA (200 μL/well) at room temperature for 1 hour.

Cells were collected from tissue culture plates with 3 mL of dissociation buffer solution (2 mmol/L EDTA, 0.05% BSA in TBS), washed with TBS, and resuspended in cell binding buffer (20 mmol/L 4‐(2‐hydroxyethyl)‐1‐piperazineethanesulfonic acid (HEPES), 150 mmol/L NaCl, 4 mg/mL BSA, pH 7.4) at ~5 × 10^5^ cells/mL. The coated plates were washed once with TBS with Ca/Mg incubated with equal amount (50 μL/well) of cell suspension and TBS with Ca/Mg for 2 hours at 37°C. Following incubation, plates were gently washed with 200 µL/well of TBS with Ca/Mg to remove nonadhered cells (or until no cells left in the lowest matrix protein coated wells, about four washing). The adhered cells were then lysed in 100 µL of lysis buffer (50 mmol/L sodium acetate pH 5.0, 1% Triton X‐100, 4 mg/mL *p*‐nitrophenyl phosphate) and incubated at 4°C overnight. The reaction was neutralized the next day by addition of 50 µL of 1 mol/L NaOH. The absorbance was determined at 405 nm using a microplate reader (Epoch; BioTek).

### 2.9 Western blot analysis

Cells (2 × 10^5^) were plated in 60‐mm tissue culture dishes and incubated with PC growth medium and 10 µmol/L 1,25(OH)_2_D_3_ or solvent control for 48 hours. Cells were then rinsed once with serum‐free DMEM and incubated with serum‐free growth medium containing appropriate treatment for 48 hours. The conditioned medium (CM) was collected and cell debris removed by 10 minutes centrifugation at 400×g. The cells on the plate were rinsed once with 0.04% EDTA in PBS and lysed with 100 µL of lysis buffer (50 mmol/L HEPES pH 7.5, 1 mmol/L MgCl_2_, 1 mmol/L CaCl_2_, 100 mmol/L NaCl, and 0.1 mmol/L EDTA with 1% NP‐40, 1% Triton X‐100, 0.5% deoxycholic acid, and protease inhibitor cocktail; Roche Biochemicals, Mannheim, Germany). Lysed cells were briefly sonicated and centrifuged at 14 000×g for 20 minutes at 4°C.

Murine VEGF165 (Cat#: 450‐32; Peprotech) and PDGF‐BB (Cat#: 315‐18; Peprotech) were used for VEGF, PDGF, and their combination treatments. For these treatments, cells were serum starved (kept in serum free medium) for 24 hours before the treatment. For the mitogen‐activated protein kinase (MAPK) activation, cells were also serum starved for 24 hours before the treatment, and the treatment was carried out in a serum‐free normal glucose medium at various time points. In some cases, total protein lysates were prepared from these samples in a slightly modified lysis buffer by addition of 2 mmol/L sodium orthovanadate and 2 mmol/L sodium fluoride. For VEGF receptor inhibition, SU4312 (S8567; Sigma‐Aldrich) was used at 100 ng/mL. For collection of total lysates, cells were not serum starved and complete medium containing appropriate treatments (48 hours) were used.

Protein and CM concentration of the collected samples were assessed using a Pierce^TM^ BCA protein assay kit (Cat#: 23225; ThermoFisher). Samples were adjusted for 50 µg protein content, and were mixed with appropriate volume of 6× SDS loading buffer and analyzed by 4%‐20% Sodium dodecyl sulfate‐Polyacrylamide gel electrophoresis (SDS‐PAGE) (Tris‐Glycine gels; Invitrogen). The proteins were then transferred to a nitrocellulose membrane and incubated in blocking buffer (5% skim milk in TBS containing 0.05% Tween 20) for at least 1 hour at room temperature followed by 2 hours incubation with appropriate primary antibody (1:1000 ratio) at room temperature or overnight at 4°C. The following antibodies were used: Anti‐VDR (Cat#: SC‐13133, D‐6; Santa Cruz), anti‐VDR (Cat#: SC‐1008, C‐20; Santa Cruz), rat anti‐mouse anti‐VEGF‐R2/FLK‐1 (Cat#: MAB443; R&D Systems), and rabbit anti‐mouse anti‐VEGF‐R2/FLK‐1 (Cat#: PA1‐21025; ThermoFisher, Rockford, IL).

The membranes were washed three times, 10 minutes each, with TBS containing 0.05% Tween 20, and then incubated with horseradish‐peroxidase‐conjugated secondary antibody (1:5000; Jackson ImmunoResearch Laboratories, West Grove, PA) for 1 hour at room temperature. Membranes were then washed four times, 10 minutes each, and visualized according to the chemiluminescent procedure using Amersham^TM^ ECL^TM^ (Chemiluminescent reagent, Cat#: RPN2209; GE Healthcare). β‐Actin was used as a loading control. For quantitation analysis, mean band intensities were measured using Imagej software (National Institute of Health, Bethesda, MD) and normalized to the β‐actin band. For MAPK and Protein kinase B (AKT) phosphorylation analysis, the phosphorylated levels were normalized to total protein levels, and then normalized to the β‐actin level. The results were reported as percentage of nontreated or solvent‐treated conditions. These experiments were repeated at least three times.

### 2.10 VEGF measurements

The effect of calcitriol and its solvent on secreted VEGF from PC was determined using a mouse VEGF Immunoassay kit (R&D Systems). Cells were plated at 2 × 10^5^ on 60‐mm tissue culture dishes; the next day, medium was changed to medium containing appropriate treatment for 48 hours. Cells were then rinsed once with serum‐free DMEM and incubated in 2 mL serum‐free PC medium for 48 hours. CM was collected and centrifuged for 10 minutes at 400×g to remove the debris, and the secreted VEGF level was determined by an enzyme‐linked immunosorbent assay enzyme‐linked immunosorbent assay (ELISA). For the cell‐associated VEGF level, cell lysate was collected and used in the ELISA assay. Fifty microliters of samples was used in the assay. The assay performed in triplicate and was normalized to the number of cells. To determine the amount of VEGF, a standard VEGF curve was prepared and used to determine VEGF concentration.

### 2.11 Statistical analysis

Statistical differences between groups were evaluated with the one‐way ANOVA, followed by the Tukey's multiple comparison test using GraphPad Prism version 5.04 for Windows (GraphPad Software, La Jolla, CA). Statistical differences were confirmed with Bonferroni's comparison of selected pairs of columns and Student's unpaired *t* test (two‐tailed). Mean ± SD is shown. A value of *P* ≤ 0.05 was considered significant.

## 3 RESULTS

### 3.1 Isolation and characterization of retinal PC

**Figure 1:**
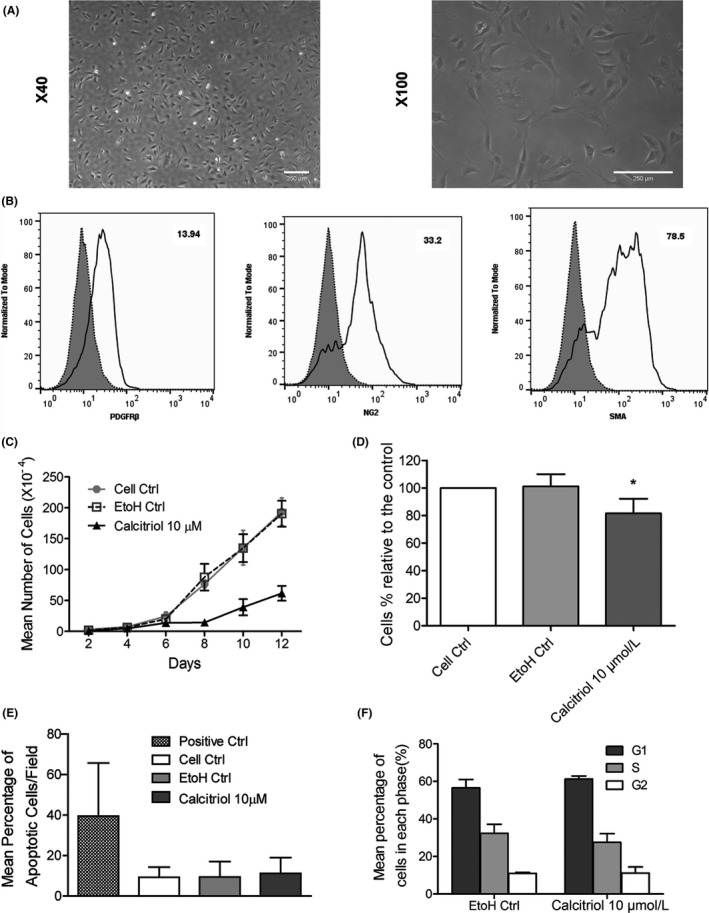
1,25(OH)_2_D_3_ (calcitriol) attenuates retinal pericyte proliferation without changes in their apoptosis. Morphology (A) and expression of known pericytes (PC) markers platelet‐derived growth factor‐Rβ, NG2, and SMA (B) are shown. The representative mean fluoresce intensities are indicated in each histogram. PC proliferation was significantly attenuated in the presence of 1,25(OH)_2_D_3_ (calcitriol) compared with solvent‐treated (ethanol; EtOH) and untreated cells (Cell Ctrl) in culture both by cell counting (C; n = 4, *P < 0.05) and rate of DNA synthesis (D; n = 4, *P < 0.05) assessments. The rate of PC apoptosis in response to calcitriol remained unchanged (E; n = 3, *P* = 0.7567). DNase I treatment was used as a positive control. A moderate increase in percentage of PC in G_1_ phase of the cell cycle was observed with 1,25(OH)_2_D_3_ treatment (F; n = 3, *P* = 0.1555 for G_1_). Scale bar = 250 µm

Retinal PC were isolated from C57BL/6j and Vdr−/− (B6.129S4‐Vdr^tm1Mbd^/J; Jackson Laboratories, Bar Harbor, ME; stock number 006133) mice crossed with Immorto mice on C57BL/6J background and genotyped as previously described.^15^ Figure 1A shows the morphology of wild‐type (WT) cells. These cells were then tested for the purity and expression of known PC markers 15, 16 such as PDGF‐Rβ, αSMA, and NG‐2^4^ by FACS analysis (Figure 1B). Comparable morphology and purity was observed in similarly prepared Vdr−/− PC (Figure S1). However, Vdr−/− PC expressed higher levels of NG2 and Smooth Muscle Actin (SMA) compared to WT cells. This is consistent with enhanced proliferation and migration of Vdr−/− PC (presented below).

### 3.2 1,25(OH)_2_D_3_ attenuates PC proliferation

Pericytes are important modulator of vascular stability and function.^4^ To examine the effect of 1,25(OH)_2_D_3_ on PC function, we first assessed cell viability after 24, 48, and 72 hours of incubation with different concentrations of 1,25(OH)_2_D_3_ (0.001‐100 µmol/L). No significant changes in cell viability in response to 1,25(OH)_2_D_3_ at concentrations equal or below 10 µmol/L were observed (not shown). We have previously used 10 µmol/L and 48 hours treatment for studies of EC incubated with 1,25(OH)_2_D_3_.^8^ Thus, we selected 10 µmol/L and 48 hours of incubation for all of the experiments performed here.

We next determined the impact of 1,25(OH)_2_D_3_ on PC proliferation. The rate of proliferation was evaluated by counting the number of cells for 2 weeks. A significant decrease in the proliferation of retinal PC incubated with 1,25(OH)_2_D_3_ was observed compared with solvent‐treated (ethanol; EtOH Ctrl) and untreated cells (Figure 1C). To assess whether the observed decrease in proliferation was due to changes in the rate of DNA synthesis, EdU labeling was utilized as described in Methods. We detected a significant decrease in the percentage of PC undergoing active DNA synthesis in the presence of 1,25(OH)_2_D_3_ (Figure 1D).

1,25(OH)_2_D_3_ studies of cancer and other cell lines have shown an increase in the rate of cell death by apoptosis.^17^, ^18^, ^19^, ^20^ However, 1,25(OH)_2_D_3_ does not always induce apoptosis.^17^, ^21^ We next asked whether retinal PC undergo apoptosis in response to 1,25(OH)_2_D_3_ and evaluated the late stage of apoptosis using a TdT‐dUTP TUNEL assay. Figure 1E shows no significant increase in the apoptosis of PC incubated with 10 µmol/L 1,25(OH)_2_D_3_ for 48 hours.

To further investigate the reason for the observed decrease in PC proliferation incubated with 1,25(OH)_2_D_3_, we assessed the PC cell cycle distribution. The percentage of cells in G_1_ phase increased in PC incubated with 1,25(OH)_2_D_3_ compared with the solvent control group (Figure 1F). Consequently, the percentage of cells in G_2_ and S phases was decreased in the 1,25(OH)_2_D_3_ group. Cell cycle arrest in different cell types in response to 1,25(OH)_2_D_3_ or other vitamin D analogs have been previously reported.^17^, ^21^, ^22^, ^23^ Thus, 1,25(OH)_2_D_3_ results in attenuation of PC proliferation by decreasing the rate of DNA synthesis and enhanced G_1_ arrest without a significant effect on apoptosis.

### 3.3 1,25(OH)_2_D_3_ attenuates retinal PC migration

**Figure 2:**
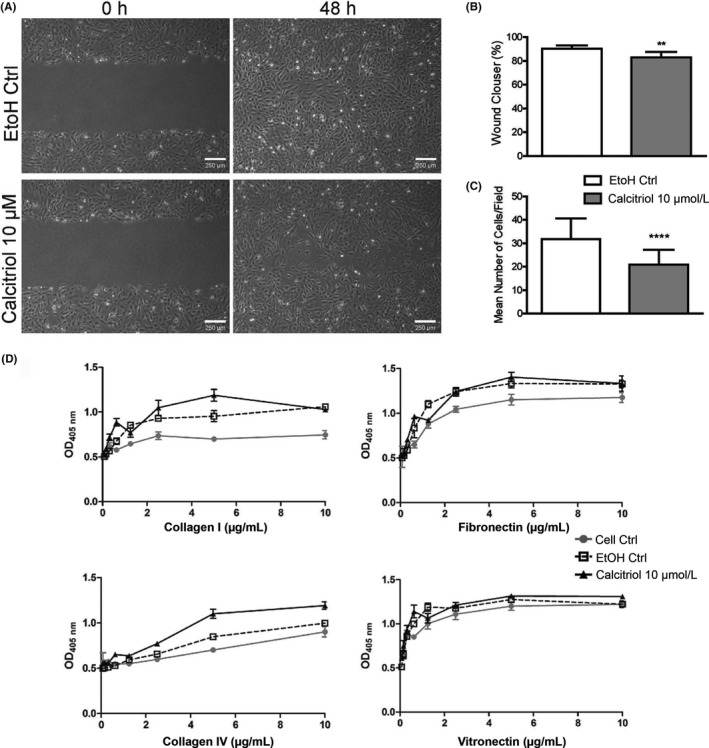
Incubation of retinal pericytes (PC) with calcitriol attenuated migration and increased their adhesion. Significant delay was observed in PC wound closure in the presence of 1,25(OH)_2_D_3_ (calcitriol) as shown in scratch wound images (A). The quantitative assessment of the data is shown in (B; n = 3, **P < 0.01). Similar results were observed in transwell migration assays (C; n = 3, ****P < 0.0001). A moderate increase in PC ability to adhere to collagen I, collagen IV, and fibronectin was observed in the presence of 1,25(OH)_2_D_3_ (Calcitriol). Scale bar = 250 µm

Pericytes migration play an important role in coverage, stabilization, and maturation of newly forming blood vessels. We assessed the migratory properties of PC in the presence of 1,25(OH)_2_D_3_ using the scratch wound and transwell migration assays. We observed a significant delay in wound closure of retinal PC incubated with 1,25(OH)_2_D_3_ (Figure 2A). Similar results were observed in the transwell migration assay. The number of cells migrated through the transwell decreased significantly in the presence of 1,25(OH)_2_D_3_ compared with solvent control (Figure 2C).

The alteration in the migratory properties of retinal PC incubated with 1,25(OH)_2_D_3_ suggested the possibility of changes in their adhesive properties. We next examined the ability of PC incubated with 1,25(OH)_2_D_3_ to adhere to various extracellular matrix (ECM) proteins, including Col I, Col IV, FN, and VN. A modest increase in adhesion to these ECM proteins was observed in retinal PC incubated with 1,25(OH)_2_D_3_ (Figure 2D).

**Figure 3:**
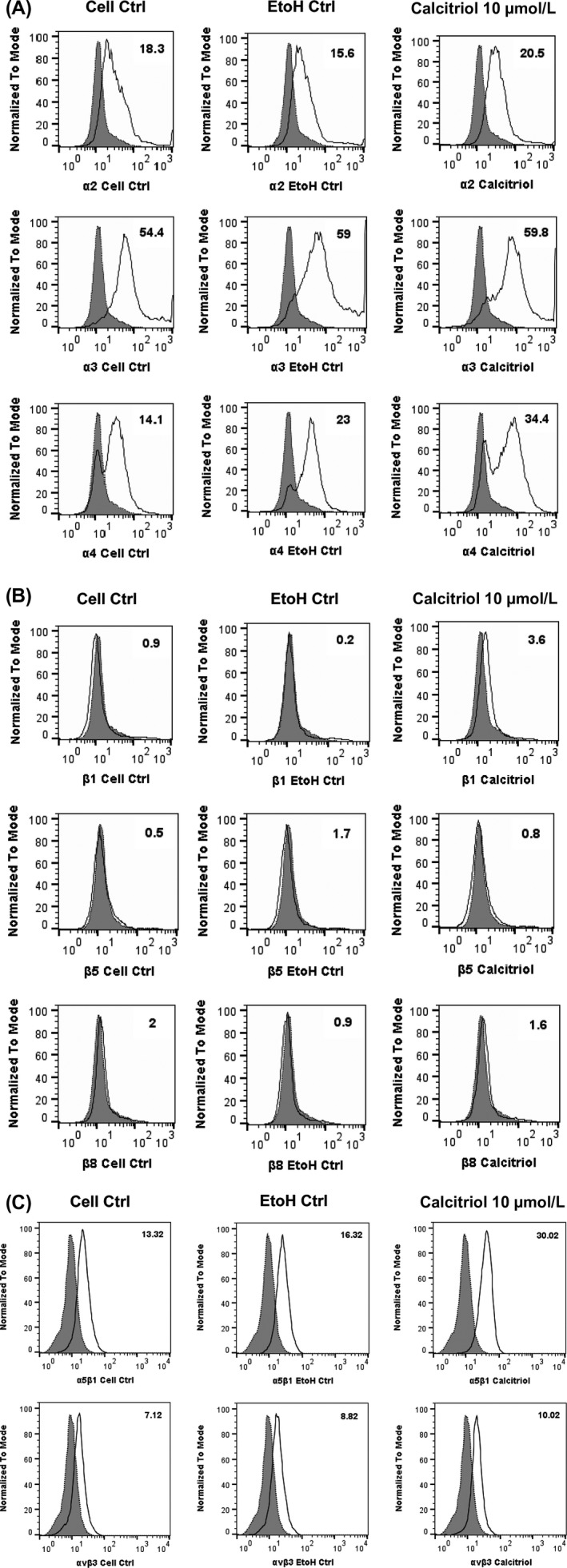
Increase in expression of α_4_ and α_5_β_1_ integrins in pericytes (PC) incubated with calcitriol. Expression levels of integrins *α*_2_, *α*_3_, *α*_4_ (A), *β*_1_, *β*_5_, *β*_8_ (B), and *α*_5_ *β*_1_, *α*_V_ *β*_3_ (C) were assessed in PC incubated with calcitriol compared with solvent‐treated (EtOH) and untreated cells using specific antibodies and FACS analysis. These experiments were repeated with two isolation of retinal PC with similar results. The representative mean fluoresce intensities are indicated in each histogram

Cell surface integrins play an important part in adhesion to ECM proteins during migration and adhesion. To determine whether observed alterations in cell migration and adhesion were associated with changes in integrin expression, we assessed cell surface expression of various integrins in PC incubated with 1,25(OH)_2_D_3_ by FACS analysis (Figure 3A‐C). Expression levels of integrins *α*_2_, *α*_3_, *β*_1_, *β*_5_, *β*_8_, and *α*_V_ *β*_3_ signified no dramatic changes in retinal PC incubated with 1,25(OH)_2_D_3_ compared with ethanol‐treated and nontreated cells. However, expression levels of integrins *α*_4_ and *α*_5_ *β*_1_ increased in PC incubated with 1,25(OH)_2_D_3_. These results are consistent with binding of the *α*_5_ *β*_1_ and *α*_4_ *β*_1_ to FN and enhanced calcification by promoting osteoblastic differentiation of vascular smooth muscle cells (VSMC) through the ERK signaling pathway and enhanced Runx2 activity.^24^, ^25^, ^26^

### 3.4 Increased VEGF production in PC incubated with 1,25(OH)_2_D_3_

**Figure 4:**
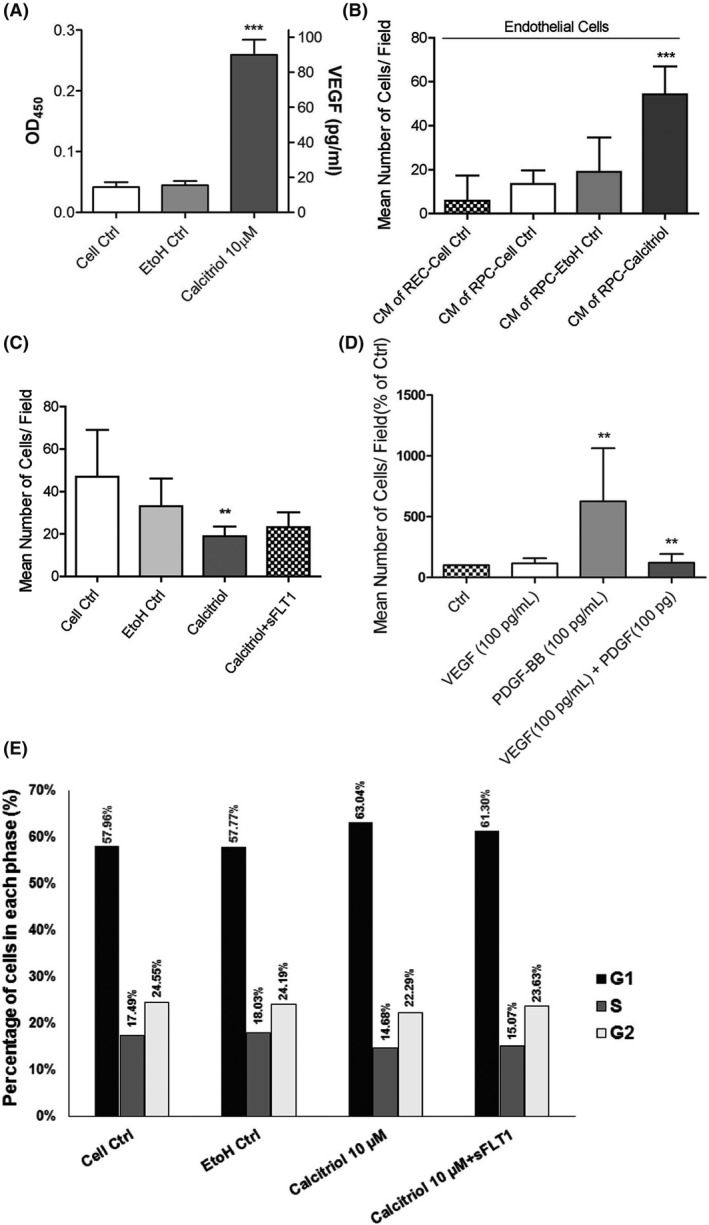
Increased vascular endothelial growth factor (VEGF) production in pericytes (PC) incubated with calcitriol. The VEGF levels were determined using an enzyme‐linked immunosorbent assay (ELISA) as detailed in Methods. A significant increase in VEGF production was observed in PC incubated with calcitriol (A; n = 3, ***P < 0.001). Endothelial cell migration increased significantly in the presence of conditioned medium collected from PC incubated with calcitriol (B; n = 3, ***P < 0.001). This observation further emphasizes the promigratory activity of the conditioned medium, likely as a result of increased secreted VEGF, from PC incubated with calcitriol. Incubation of PC treated with calcitriol in the presence of VEGF trap (sFLT‐1) restored PC migration (C; n = 3, **P < 0.01). Thus, increased VEGF level is partially responsible for inhibition of PC migration incubated with calcitriol. Similarly, the observed cell cycle arrest in G_0_/G_1_ phase of PC incubated with calcitriol was partially restored by sFLT‐1 (D). The effect of VEGF and platelet‐derived growth factor (PDGF) combination on PC migration was evaluated by transwell migration (E; n = 3, **P < 0.01). Incubation of PC with VEGF attenuated their PDGF‐mediated migration

Vascular endothelial growth factor is an important modulator of angiogenesis. During vessel formation, PC secrete VEGF to promote EC survival. We measured secreted VEGF levels in retinal PC incubated with 1,25(OH)_2_D_3_ or solvent control. We observed a significant increase in the amount of VEGF produced by PC incubated with 1,25(OH)_2_D_3_ (Figure 4A). We also found that the majority of the detected VEGF is in the secreted form and not cell associated (Figure S2). To determine whether the secreted VEGF by PC incubated with 1,25(OH)_2_D_3_ affects retinal EC function, we examined the effects of medium conditioned by PC on EC function, as previously reported by others.^27^, ^28^ We determined the effect of CM from PC incubated with 1,25(OH)_2_D_3_ or solvent control on retinal EC migration. We observed a significant increase in migration of retinal EC incubated with the CM prepared from 1,25(OH)_2_D_3_ incubated PC (Figure 4B). These results are consistent with increased production of VEGF by PC incubated with 1,25(OH)_2_D_3_ and enhanced EC migration.

### 3.5 VEGF antagonism reversed 1,25(OH)_2_D_3_‐mediated inhibition of PC migration and cell cycle arrest

To assess whether decreased migration of PC incubated with 1,25(OH)_2_D_3_ is due to increased VEGF production, we used the VEGF trap (sFLT‐1) to antagonize VEGF effects. sFLT‐1 has a VEGF binding domain of FLT‐1 and also a 31‐amino‐acid stretch derived from an intron,^29^ a protein that tightly binds VEGF and prevents its angiogenic activity. sFLT‐1 (100 ng/mL) partially reversed the inhibition of PC migration by 1,25(OH)_2_D_3_ (Figure 4C). A modest reversal was also observed in cell cycle arrest at G_1_ phase (Figure 4D). The percentage of cells observed in G_1_ phase was similar between nontreated (cell control), solvent control (ethanol), and those incubated with sFLT‐1. The modest restoration of migration and cell cycle arrest could be explained by the fact that sFLT‐1 cannot trap all forms of secreted VEGF.

During angiogenesis, EC secrete PDGF to recruit PC containing PDGF‐Rβ to the nascent vascular sprouts and stabilize the newly forming blood vessels. We next measured the effect of VEGF (100 pg/mL; close to the secreted VEGF levels in PC incubated with 1,25(OH)_2_D_3_), PDGF, and combination of both as a disruptive proposed mechanism by Greenburg et al.^7^ Incubation of PC with VEGF did not affect PC migration (Figure 4E). However, a combination of VEGF and PDGF attenuated PDGF‐mediated PC migration (Figure 4E). Thus, the presence of exogenous VEGF has a pivotal role in inhibiting PC migration by PDGF and favoring a stabilized and quiescent phenotype.

### 3.6 Expression of VEGF receptors in retinal PC

**Figure 5:**
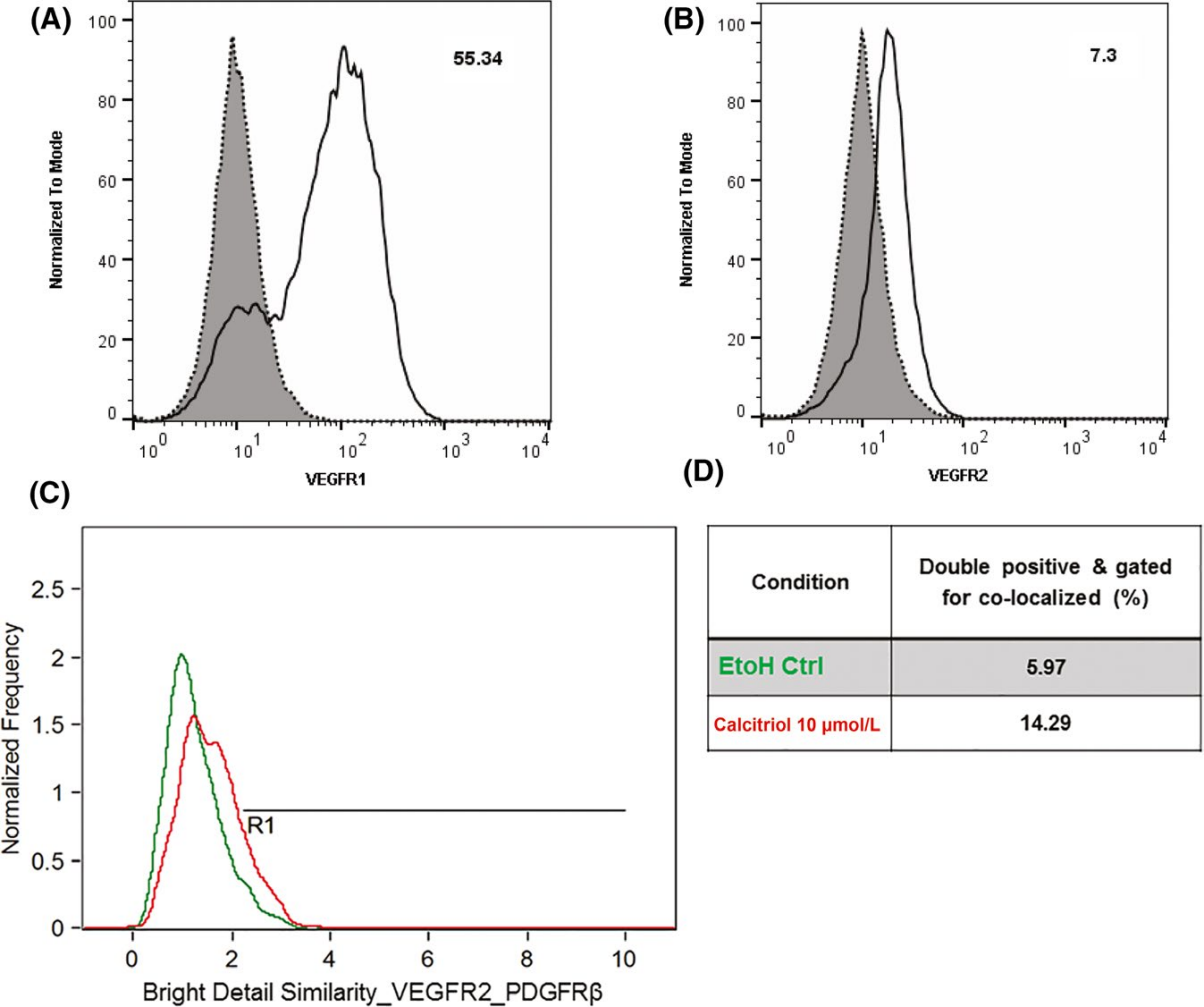
Expression of vascular endothelial growth factor (VEGF) receptors in retinal pericytes (PC). PC express VEGF‐R1 (A) and VEGF‐R2 (B). The histogram distribution of cells incubated with solvent control (ethanol, EtOH) or calcitriol for 48 h and stained with anti‐platelet‐derived growth factor (PDGF)‐Rβ and VEGF‐R2 (C). A shift to the right indicating increased costaining for both receptors in calcitriol‐treated cells was observed. Colocalization of VEGF‐R2 and PDGF‐Rβ increased by approximately 2.5‐fold in PC incubated with calcitriol compared with solvent control and quantifications are shown in (D). R1 indicates the shift in fluorescence intensity

Based on the observed effects of 1,25(OH)_2_D_3_ on VEGF, and attenuation of PDGF‐mediated PC migration by VEGF, we next determined the expression of VEGF receptors in PC. Retinal PC expressed both VEGF‐R1 and VEGF‐R2 on their surface (Figure 5A,B). Studies of Greenberg et al defined a new role for VEGF in PC. VEGF expression can negatively affect the proangiogenic function of PC through enhanced heterodimerization of VEGF‐R2 and PDGF‐Rβ, and attenuation of signaling through both receptors by their respective ligands on PC.^7^ To delineate whether the observed angioinhibitory function of 1,25(OH)_2_D_3_ is through heterodimerization of VEGF‐R2 and PDGF‐Rβ on PC, we tested their colocalization/proximity by Amnis imaging flow cytometry analysis. This technology provides an improved method of studying colocalization through combination of imaging and flow cytometry in individual cells.^30^ Mouse retinal PC showed approximately a threefold increase in the percentage of the cells that expressed both receptors along with their colocalization, demonstrating the potential heterodimerization of VEGF‐R2 and PDGF‐Rβ on PC incubated with 1,25(OH)_2_D_3_ (Figure 5C,D), as previously demonstrated.^7^

### 3.7 Alteration in intracellular signaling pathways of retinal PC in response to 1,25(OH)_2_D_3_, or VEGF and PDGF

**Figure 6:**
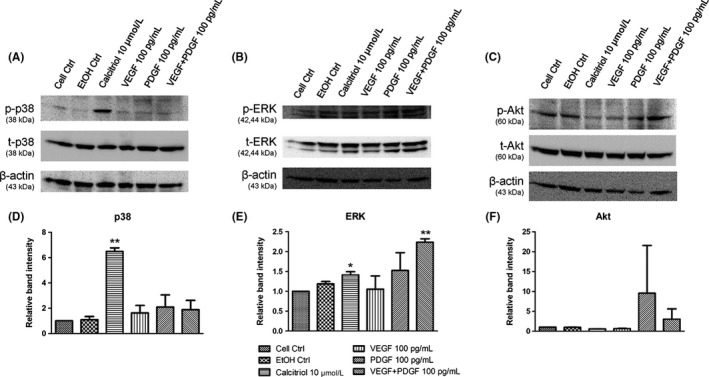
Alterations in intracellular signaling pathways of retinal pericytes (PC) in response to 1,25(OH)_2_D_3_ (calcitriol) or vascular endothelial growth factor (VEGF) and platelet‐derived growth factor (PDGF). Effect of calcitriol, VEGF, and VEGF + PDGF on mitogen‐activated protein kinases (MAPK) activation proteins expression and activity of P38 (A), ERK (B), and AKT (C) in PC is examined using Western blot analysis. Quantifications are shown (D‐F; n = 3), respectively, using Imagej analysis. Increased activation of P38 by calcitriol (**P < 0.01) but not VEGF, PDGF, or VEGF and PDGF was observed. In contrast, ERK was significantly activated by both calcitriol and combination of VEGF and PDGF (*P < 0.05; **P < 0.01). The AKT activation was suppressed by calcitriol. Although PDGF increased AKT activation in PC, this was suppressed when PC incubated with PDGF and VEGF. These differences did not reach significant levels

The family of MAPK and AKT signaling pathways are involved in the regulation of diverse range of cellular processes including 1,25(OH)_2_D_3_ mediated VSMC osteoblastic differentiation and calcification.^13^, ^26^, ^31^, ^32^, ^33^, ^34^ Here, we examined the effect of 1,25(OH)_2_D_3_, VEGF, and VEGF and PDGF on MAPK protein expression and activity including p38 and extracellular‐signal‐regulated kinases (ERK), and AKT in PC (Figure 6). We first assessed the temporal expression of these proteins (1, 12, and 24 hours) and observed the activation as soon as 1 hour after incubation with 1,25(OH)_2_D_3_, which was sustained for up to 24 hours (not shown). Here, we used overnight serum starvation and incubation with 1,25(OH)_2_D_3_ for 24 hours for the assessment of activation status of MAPK and AKT proteins.

Growth factors, environmental stress, and inflammatory cytokines can activate p38.^33^, ^35^, ^36^ Hedges et al demonstrated the involvement of p38 in VSMC migration.^37^ Here, we showed that incubation of PC with 1,25(OH)_2_D_3_ activates p38. This is consistent with the reported role of p38 activation in VSMC calcification.^32^, ^33^ However, VEGF, PDGF, or their combination did not activate p38 (Figure 6A,D). The ERK pathway regulates cell proliferation, apoptosis, cell cycle arrest, and adhesion. In PC, ERK regulates survival pathways through the activation of PDGF‐Rβ.^38^ Our data indicated sustained activation of ERK in PC incubated with 1,25(OH)_2_D_3_, which is consistent with the proposed role of ERK in VSMC calcification through modulation of Runx2 activity.^26^, ^31^ Although, in retinal PC, no significant effect on ERK activation was observed by VEGF (100 pg/mL), the combination of VEGF and PDGF, similar to 1,25(OH)_2_D_3_, activated ERK (Figure 6B,E). Thus, the inhibition of PC proliferation and migration by attenuation of signaling through VEGF‐R2 and PDGF‐Rβ by 1,25(OH)_2_D_3_ or combination of VEGF and PDGF are concomitant with the activation of ERK, and increased expression of *α*_5_ *β*_1_ and enhanced adhesion to FN.^26^

The AKT pathway is both proangiogenic and antiangiogenic, and it controls cell survival, cell cycle, cell proliferation, and migration.^39^, ^40^ AKT signaling also plays an important role in VSMC calcification.^32^, ^33^ In retinal PC, 1,25(OH)_2_D_3_ decreased AKT activation. Similarly, VEGF suppressed PDGF‐mediated AKT activation in PC (Figure 6C,F). Thus, these results indicated that incubation of retinal PC with 1,25(OH)_2_D_3_ results in p38 and ERK activation. The combination of VEGF and PDGF also caused ERK, but not p38, activation in PC. Although AKT was stimulated by PDGF, combination of VEGF and PDGF suppressed its activation, as did 1,25(OH)_2_D_3_. Our results suggest that the activation of ERK and inhibition of AKT activation by 1,25(OH)_2_D_3_ are consistent with lack of signaling by VEGF in the presence of PDGF. However, 1,25(OH)_2_D_3_‐mediated p38 activation was independent of VEGF‐mediated attenuation of PDGF signaling, and likely VDR independent since the inhibition of PC migration by 1,25(OH)_2_D_3_, was impacted by p38 activity,^37^ and observed in both WT and Vdr−/− PC. This may be linked to increased oxidative stress by incubation with 1,25(OH)_2_D_3_.^41^

### 3.8 Increased VDR expression in retinal PC incubated with 1,25(OH)_2_D_3_

**Figure 7:**
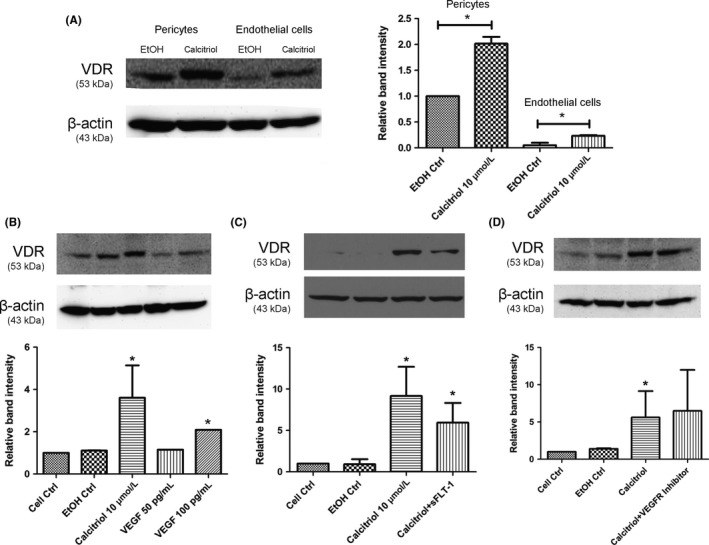
Increased vitamin D receptor (VDR) expression in retinal pericytes (PC) incubated with 1,25(OH)_2_D_3_ (calcitriol). The expression VDR was evaluated by Western blot analysis of cell lysates prepared from retinal PC and endothelial cells (EC). PC expressed higher level of VDR compared with EC, and their level increased significantly in response to calcitriol (A; n = 3, *P < 0.05). The effect of vascular endothelial growth factor (VEGF) on PC VDR expression was also assessed by Western blotting (B). Approximately, a twofold increase in VDR levels was observed in PC incubated with VEGF. VEGF trap (sFLT‐1), decreased VDR expression in PC incubated with calcitriol (C), and VEGF‐R2 inhibitor (consequently excess amount of VEGF in culture) further increased calcitriol‐mediated VDR expression (D); *P < 0.05. Thus, VEGF signaling could have a direct impact on VDR expression

The majority of vitamin D action is mediated through its receptor,^42^, ^43^ and 1,25(OH)_2_D_3_ increases VDR expression in various cell types.^42^, ^44^, ^45^, ^46^, ^47^ To further explain the observed inhibitory effect of 1,25(OH)_2_D_3_ on PC, we examined VDR expression in PC. PC expressed higher basal levels of VDR compared with EC, and its level increased significantly in the presence of 1,25(OH)_2_D_3_ (Figure 7A). VDR mRNA expression level also increased significantly in PC incubated with 1,25(OH)_2_D_3_ determined by real‐time quantitative polymerase chain reaction (qPCR) analysis (Figure S3). These results are also consistent with increased adhesion of PC incubated with 1,25(OH)_2_D_3_ to various ECM proteins, since cell adhesion is an important modulator of VDR expression.^48^

### 3.9 Effect of VEGF, VEGF antagonism, and VEGF‐R2 inhibitor on VDR expression

To assess the direct effect of VEGF on retinal PC, we incubated PC with various concentrations of mouse recombinant VEGF (50 pg/mL‐100 ng/mL). Both wound and transwell migration experiments demonstrated no significant changes in PC migration incubated with VEGF (not shown). We then assessed the effects of VEGF on VDR expression using western blot analysis. Our results showed that although VEGF increased VDR expression, it did not reach the levels observed following incubation with 1,25(OH)_2_D_3_ (Figure 7B).

We next assessed the effect of VEGF antagonism (VEGF trap; sFLT‐1) and the presence of excess VEGF signaling (VEGF‐R2 inhibitor) on VDR expression in retinal PC in the presence of 1,25(OH)_2_D_3_. VDR expression was decreased by antagonism of VEGF and increased by exogenous of VEGF in culture (Figure 7C,7). Thus, VEGF and VEGF signaling has a direct impact on VDR expression in PC, perhaps through enhanced adhesion and attenuation of VEGF‐R2 signaling.

### 3.10 1,25(OH)_2_D_3_ did not inhibit the proliferation of Vdr−/− PC

Because of increased VDR expression in retinal PC incubated with 1,25(OH)_2_D_3_, we assessed whether the observed angioinhibitory properties of 1,25(OH)_2_D_3_ in PC are mediated through VDR. We initially knockdown VDR levels in retinal PC using shRNA lentivirus particles and assessed the impact of 1,25(OH)_2_D_3_ on PC proliferation. Although we successfully knockdown VDR levels, incubation of control and knockdown cells with 1,25(OH)_2_D_3_ resulted in significant upregulation of VDR expression, as occurs in other cell types, making it difficult to assess the contribution of VDR independent activity to PC function. Thus, we isolated PC from Vdr−/− mice for these studies. In addition to PC markers presence as shown in Figure S1, the presence of VEGF receptors in Vdr−/− PC was confirmed by FACS analysis (Figure S4).

**Figure 8:**
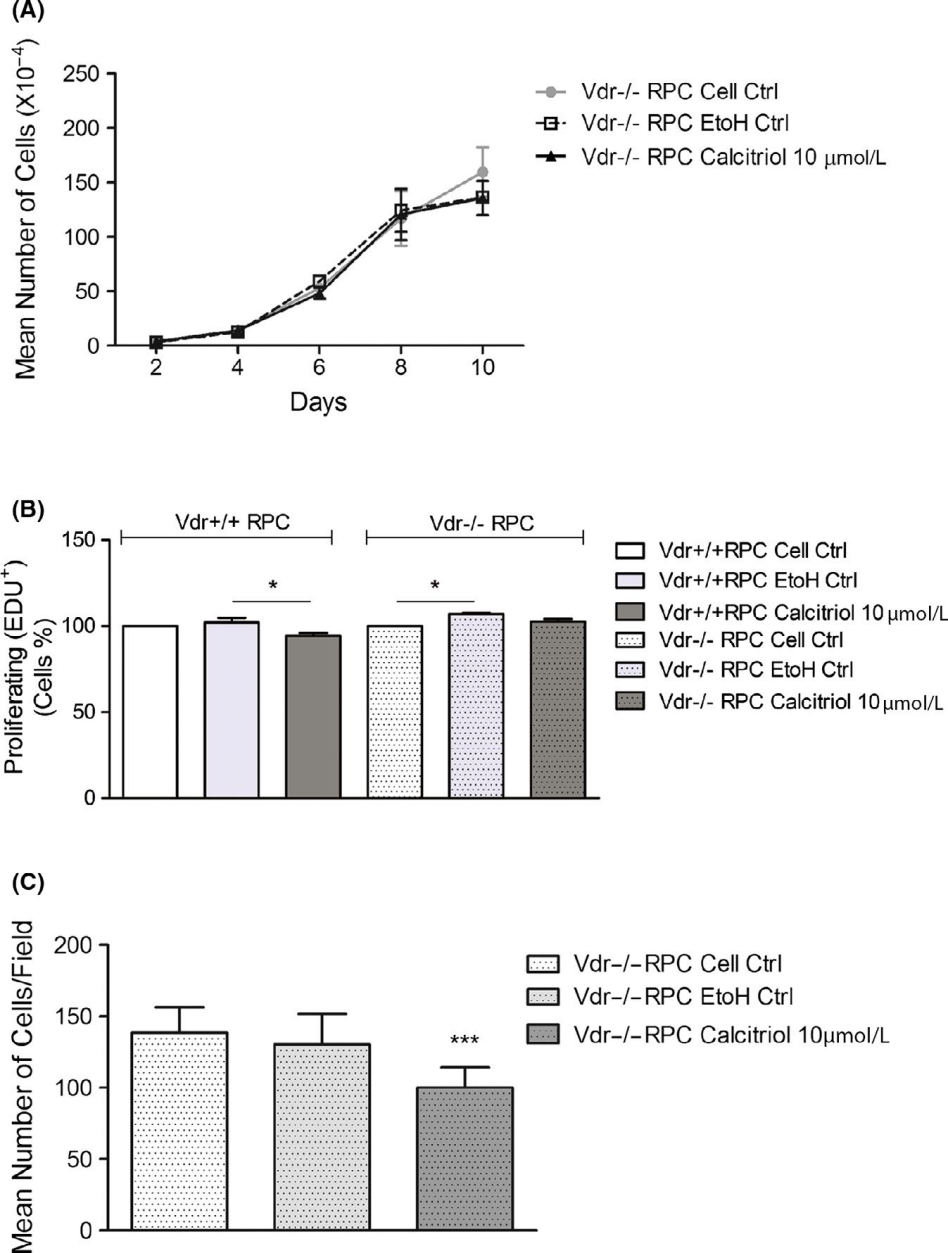
1,25(OH)_2_D_3_ (calcitriol)‐mediated inhibition of pericytes (PC) proliferation is vitamin D receptor (VDR) dependent, while the inhibition of migration is not. The impact of calcitriol on proliferation Vdr−/− PC was assessed as was done with wild type cells. Vdr−/− retinal PC proliferation was not affected by calcitriol (A; n = 3, *P* = 0.3787). Similar results was observed by assessing rate of DNA synthesis (B; n = 3, *P < 0.05). Thus, inhibition of PC proliferation by calcitriol is VDR dependent. In contrast, the inhibition of PC migration was independent of VDR expression. Incubation of Vdr−/− retinal PC with calcitriol significantly decreased their migration (C; n = 3, ***P < 0.001)

We next determined the rate of proliferation in Vdr−/− retinal PC. incubated with 1,25(OH)_2_D_3_ as described above for Vdr+/+ retinal PC Vdr−/− PC grew slightly faster than the Vdr+/+ PC, and the rate of Vdr−/− PC proliferation, unlike Vdr+/+ PC, was not affected by the presence of 1,25(OH)_2_D_3_ (Figures 1C and 8A). To assess the rate of DNA synthesis, Vdr−/− PC were incubated with 1,25(OH)_2_D_3_ and compared with Vdr+/+ PC using EdU labeling as described in Methods. We detected a decrease in the percentage of PC undergoing active DNA synthesis in Vdr+/+ PC incubated with 1,25(OH)_2_D_3_ as showed above (Figure 1D). However, 1,25(OH)_2_D_3_ did not impact Vdr−/− PC proliferation (Figure 8B). A modest increase in the percentage of cells at G_1_ phase of the cell cycle was observed in Vdr−/− PC incubated with 1,25(OH)_2_D_3_ (not shown). To further investigate whether the absence of VDR negated other angioinhibitory effects of 1,25(OH)_2_D_3_, we performed a transwell migration assay with Vdr−/− PC incubated with 1,25(OH)_2_D_3_. Interestingly, despite the absence of VDR, 1,25(OH)_2_D_3_ inhibited retinal PC migration (Figure 8C). Thus, the inhibition of retinal PC migration by 1,25(OH)_2_D_3_ is VDR independent.

Alteration in migratory properties of Vdr−/− retinal PC in response to 1,25(OH)_2_D_3_ suggested changes in their adhesive properties may exist. We next examined the Vdr−/− PC adhesion to various ECM proteins including FN, Col I, VN, and Col IV in the presence of 1,25(OH)_2_D_3_ (Figure S5). Vdr−/− retinal PC were more adherent to the ECM proteins compared with Vdr+/+ PC. However, adhesion of Vdr−/− PC to the ECM proteins remained unchanged in response to 1,25(OH)_2_D_3_, with the exception of moderately increased adhesion on Col I.

## 4 DISCUSSION

The potential antiangiogenic activity of vitamin D has been considered in a number of studies.^1^, ^49^, ^50^, ^51^ We have shown that 1,25(OH)_2_D_3_ inhibits retinal neovascularization during OIR in a VDR‐dependent manner.^8^, ^9^ However, the detailed mechanisms and the cellular targets involved remained poorly understood. Our previous studies showed that incubation with 1,25(OH)_2_D_3_ minimally affects the proliferation and migration of retinal EC in culture.^8^, ^52^ Here, we assessed the impact of 1,25(OH)_2_D_3_ on retinal PC function and determined whether VDR is involved. We showed that 1,25(OH)_2_D_3_ inhibits retinal PC migration and proliferation without a significant effect on their apoptosis. Although we observed a cell cycle arrest in PC incubated with1,25(OH)_2_D_3_, this did not reach a significant level. Our results are consistent with previous studies showing the inhibition of vascular and airway SMC proliferation by 1,25(OH)_2_D_3_.^53^, ^54^ In contrast, other studies reported enhanced VSMC proliferation by vitamin D,^55^, ^56^, ^57^ particularly in quiescent cells.^58^ The reasons for these contrasting results remain unclear and could be contributed to the developmental stage or sources of cells.

Adhesion of PC to various ECM proteins was moderately increased in retinal PC incubated with 1,25(OH)_2_D_3_. The expression of integrins in retinal PC incubated with 1,25(OH)_2_D_3_ showed no dramatic differences except for *α*_4_ and *α*_5_ *β*_1_ integrins, whose expression was increased. Integrin *α*_4_ plays an important role during mouse skeletal muscle development,^59^ and its deficiency results in formation of defective blood vessel and PC distribution in the mouse embryos.^60^ In addition, integrin *α*_4_ is suggested to be a PC marker, and it is expressed in the PC of newly formed vessels.^60^ Thus, the increased expression of integrin *α*_4_ in the presence of 1,25(OH)_2_D_3_ is consistent with the mature PC phenotype. Integrin *α*_5_ *β*_1_ is a predominant fibronectin receptor, and its increased expression in PC incubated with 1,25(OH)_2_D_3_ is consistent with their increased adhesion to fibronectin. In addition, the increased expression of *α*_5_ *β*_1_ is observed during differentiation of mesenchymal stem cells to PC.^61^, ^62^ Further, *α*_5_ *β*_1_ expression promotes the switch from a contractile to a synthetic phenotype in VSMC,^61^, ^63^ and it is important in PDGF‐Rβ signaling.^61^, ^64^ How 1,25(OH)_2_D_3_ causes the increased expression of *α*_5_ *β*_1_ integrin remains elusive, and may be linked to induction of calcification as demonstrated in VSMC through α_5_β_1_/ERK/Runx2 pathway.^26^, ^31^ The interaction between Runx2 and VDR modulates the expression of many genes affecting various cellular functions including adhesion and migration.^13^, ^36^, ^65^, ^66^, ^67^ However, the direct role of VDR and its potential interactions with other coregulatory factors that may affect integrin expression is complex and needs further investigation.

We observed increased VEGF secretion in PC incubated with 1,25(OH)_2_D_3_, and the majority of measured VEGF was in the secreted form and not cell associated. Increased VEGF levels is also reported in VSMC incubated with 1,25(OH)_2_D_3_. Cardus et al indicated the increased VEGF levels by 1,25(OH)_2_D_3_ could be explained by the presence of a VDRE in the VEGF promoter.^68^ To determine whether VEGF plays a role in the observed inhibitory effect of 1,25(OH)_2_D_3_ on retinal PC proliferation and migration, VEGF trap (sFLT‐1) was used to antagonize VEGF. sFLT‐1 partially reversed the 1,25(OH)_2_D_3_‐mediated decrease in migration, without a significant effect on the retinal PC proliferation. VEGF receptors are expressed in SMC.^69^ We also detected the presence of VEGF‐R1 and VEGF‐R2 in retinal PC. Thus, the observed effects of 1,25(OH)_2_D_3_ on retinal PC function could be mediated, at least in part, through interaction of VEGF with its receptors in an autocrine fashion.

Greenberg et al^7^ demonstrated that VEGF acting in an autocrine fashion could negatively regulate PC proangiogenic activity. They showed that this was mediated through heterodimerization of VEGF‐R2 and PDGF‐Rβ on PC resulting in attenuation of the proangiogenic activity of retinal PC. In tumor studies, targeting both VEGF‐R2 and PDGF‐Rβ by SU6668 leads to tumor blood vessels regression by 40%, and induced EC apoptosis.^70^ Disruption of PDGF/PDGF‐Rβ signaling in transgenic mice resulted in failure of PC recruitment to newly formed blood vessels. This was also associated with increased capillary diameter, abnormal EC shape, endothelial hyperplasia, and abnormal blood vessel maturation and stabilization.^70^, ^71^, ^72^ In addition, during retinal vascular development, disruption of already established EC and PC is observed by intraocular injection of PDGF‐BB and interfering with platelet‐derived growth factor/PDGF receptor beta (PDGF/PDGFRβ) signaling.^73^ We showed that incubation of PC with VEGF attenuated PDGF‐mediated migration. In addition, our data indicated an increase in colocalization of VEGF‐R2 and PDGF‐Rβ in retinal PC incubated with 1,25(OH)_2_D_3_. VEGF produced by PC is also involved in modulating increased permeability of blood‐brain barrier during a stroke.^74^ Thus, an optimal level for VEGF and PDGF is required for proper formation of blood vessels, and any changes in their levels or interference with their signaling could result in abnormal blood vessel formation, remodeling, or inhibition.

The MAPK family of proteins including ERKs and p38, and AKT signaling pathways are involved in regulation of diverse range of cellular processes including 1,25(OH)_2_D_3_‐mediated VSMC osteoblastic differentiation and calcification.^26^, ^31^, ^32^, ^33^, ^34^ We showed incubation of retinal PC with 1,25(OH)_2_D_3_ resulted in p38 and ERK activation. The combination of VEGF and PDGF also showed ERK, but not p38, activation. Although AKT was activated by PDGF, combination of VEGF and PDGF suppressed this activation, as did incubation with 1,25(OH)_2_D_3_. Growth factors, environmental stress, and inflammatory cytokines can activate p38.^35^ Hedges et al demonstrated the involvement of p38 activity in VSMC migration.^37^ The ERK pathway regulates cell proliferation, apoptosis, cell cycle arrest, and adhesion. In PC, ERK regulates survival pathways through the activation of PDGF‐Rβ.^38^ Here, our data demonstrated sustained activation of ERK in retinal PC incubated with 1,25(OH)_2_D_3_. Induction of VEGF also activates p38, ERK, jun‐N‐terminal kinase (JNK), and AKT through the activation of Src in primary human PC,^74^ and activates ERK in human VSMC.^7^ Although, no significant effect was observed by 100 pg/mL of VEGF in retinal PC, a combination of VEGF and PDGF increased ERK activation similar to that observed with 1,25(OH)_2_D_3_ incubation. This could indicate that the activation of ERK in PC is most likely through induction of *α*_5_ *β*_1_ 75 by its fibronectin ligand which was increased in the presence of 1,25(OH)_2_D_3_. In addition, the activation of ERK is required for S phase entry by growth factors.^76^, ^77^ Since 1,25(OH)_2_D_3_ could cause cell cycle arrest, this may result in sustained ERK activation stimulating cell cycle progression and entrance to S phase. Furthermore, sustained ERK activation could have a negative effect on the cell cycle by promoting accumulation of cell cycle inhibitors.^77^ Thus, changes in ERK activity could promote cell proliferation or growth arrest,^77^ and need further exploration.

The AKT signaling pathway has both pro‐ and antiangiogenic effects, and it controls cell survival, cell cycle, cell proliferation, and migration.^39^, ^40^ PDGFR signaling activates the AKT pathway.^38^, ^78^ In retinal PC, 1,25(OH)_2_D_3_ decreased AKT activation, consistent with the suppression of PDGF‐mediated AKT activation in the presence of exogenous VEGF. Increase oxidative stress by 1,25(OH)_2_D_3_ could directly activate p38 in VSMC impacting their calcification.^32^, ^41^ Thus, our results suggest that the activation of ERK and inhibition of AKT activation by 1,25(OH)_2_D_3_ involves the disruption of signaling mediated by VEGF and PDGF, but not p38 activation.

Although, we observed a 2.5‐fold increase in the VEGF‐R2 and PDGF‐Rβ colocalization in retinal PC incubated with 1,25(OH)_2_D_3_, we did not detect any colocalization in untreated cells. Higher levels of VDR detected in retinal PC compared with EC, and its significant increase following incubation with 1,25(OH)_2_D_3_, as well as its suppression by sFLT‐1 support the notion that retinal PC are a major target of vitamin D action and regulation during angiogenesis. This is further supported by the fact that the incubation of PC with a VEGF‐R2 inhibitor increased VDR expression to levels even greater than that with 1,25(OH)_2_D_3_ alone. Interestingly, VDR level was also increased when PC were incubated with VEGF. The increased VDR expression is consistent with increased adhesion of PC incubated with 1,25(OH)_2_D_3_ or VEGF.^48^ In addition, a functional VDR response element has been previously reported in the avian integrin *β*_3_ promoter.^79^ Thus, the inhibition of VEGF‐R2 signaling could attenuate angiogenesis through increased adhesion and production of VDR, and attenuation of proangiogenic activity of both EC and PC. These findings could be explained by the results of EVEX database^80^ search bringing separate studies that show Runx2 is a regulator of VDR^81^, ^82^ and VEGF^83^, ^84^, ^85^, ^86^, ^87^, ^88^, ^89^ expression. Thus, a direct relationship exists between VDR and VEGF expression perhaps modulated by Runx2 activity through the *α*_5_ *β*_1_/ERK pathway.^26^, ^31^ However, whether this would require that integrin *β*_1_ and/or *α*_5_ contain a VDR binding site for interaction and/or regulation by Runx2 remains to be determined. To our knowledge, this is the first report demonstrating the effect of VEGF/VEGF receptor signaling on VDR expression and activity.

As the majority of vitamin D action is believed to be mediated through its receptor (VDR), we isolated retinal PC from Vdr−/− mice and determined the effect of 1,25(OH)_2_D_3_ on these cells. Our data demonstrated that Vdr−/− retinal PC were more proliferative, migratory, and adhesive compared to Vdr+/+ PC. In addition, Vdr−/− PC expressed VEGF‐R1 and ‐R2. 1,25(OH)_2_D_3_ failed to inhibit the proliferation of Vdr−/− PC. However, migration of Vdr−/− PC was affected by 1,25(OH)_2_D_3_ with a modest change in their adhesive properties. A minimal increase in G_1_ phase of the cell cycle was also observed in Vdr−/− PC incubated with 1,25(OH)_2_D_3_. These novel findings suggest important roles for VDR in retinal PC response to 1,25(OH)_2_D_3_, and will allow the identification VDR‐independent pathways affecting PC function.

**Figure 9:**
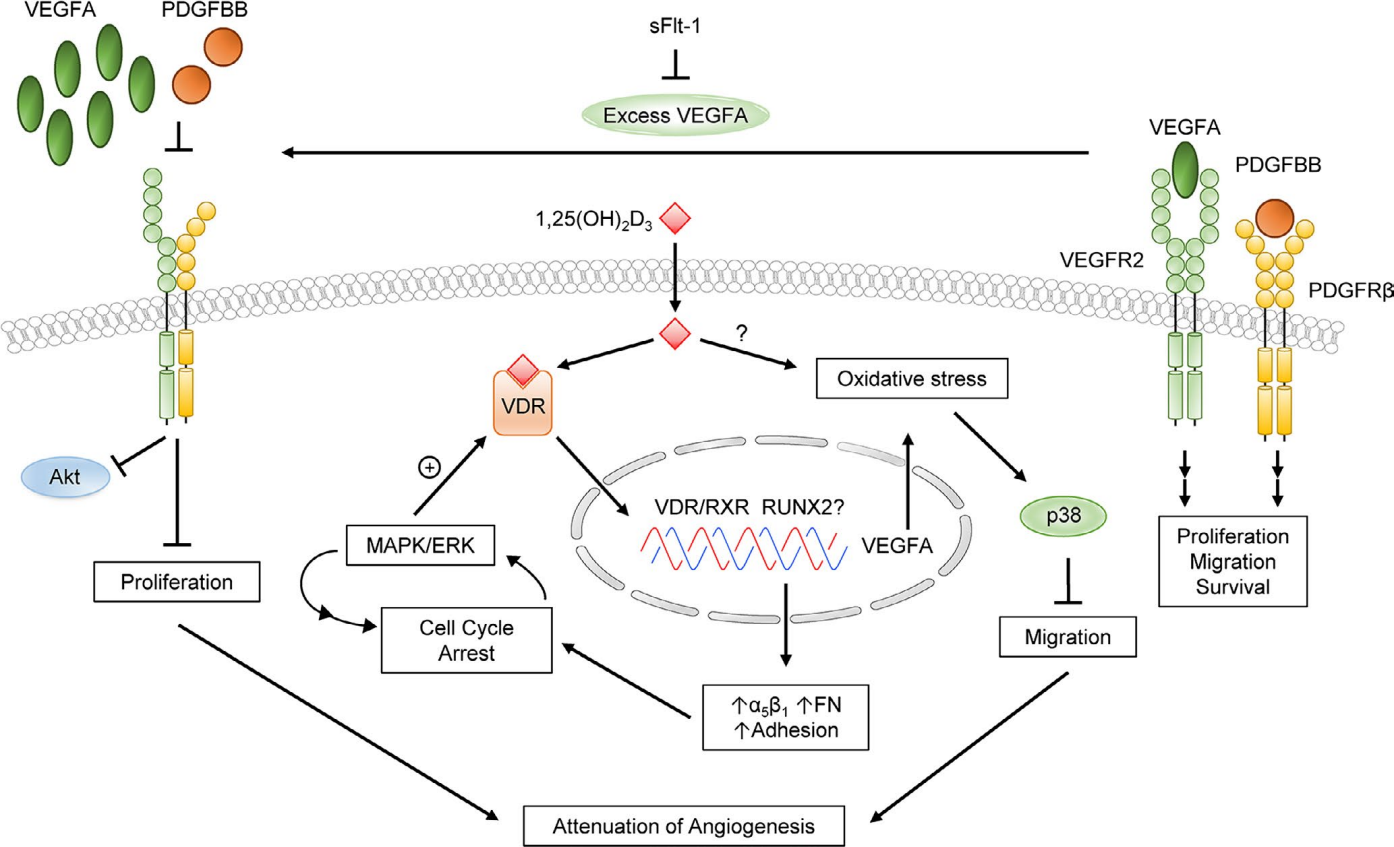
1,25(OH)_2_D_3_ (calcitriol)‐mediated inhibition of pericytes (PC) proliferation and migration. 1,25(OH)_2_D_3_ inhibits PC proliferation though increased production of vascular endothelial growth factor (VEGF), which promotes heterodimerization of VEGF receptor‐2/FLK‐1 (VEGFR2) and PDGFRβ. This results in attenuation of signaling though these receptors and attenuating PC proliferation. In addition, 1,25(OH)_2_D_3_ enhances p38 activation, likely through increased oxidative stress in a VDR‐independent manner attenuating PC migration. In addition, the increased expression of the extracellular matrix protein fibronectin (FN) and its receptor α5β1 integrin is consistent with increased adhesion, mitogen‐activated protein kinases (MAPK)/ERK activation, and cell cycle arrest in wild‐type PC

In summary, our studies defines PC as a target of 1,25(OH)_2_D_3_ in the vasculature and suggest that the angioinhibitory function of 1,25(OH)_2_D_3_ in the retina could be mediated through the inhibition of PC proangiogenic activity including proliferation and migration (Figure 9). We demonstrated that these observed effects in PC could be mediated, in part, through VDR, increased production of VEGF, and consequently, the attenuation of signaling through PDGF‐Rβ and VEGF‐R2 in PC promoting their quiescent phenotype.

## CONFLICT OF INTEREST

The authors declare that there are no competing interests associated with the manuscript.

## AUTHOR CONTRIBUTIONS

N. Sheibani and N. Jamali conceived and designed the experiments. N. Jamali, Y.‐S. Song, and CM Sorenson resourced and performed the experiments. NJ and N. Sheibani analyzed the data. N. Jamali, Y.‐S. Song, CM Sorenson, and N. Sheibani wrote and edited the manuscript.
