# Supplementary material for: 1,25(OH)2D3 regulates the proangiogenic activity of pericyte through VDR‐mediated modulation of VEGF production and signaling of VEGF and PDGF receptors
Source: FASEB Bioadv. 2019 May 21;1(7):415–34. doi: 10.1096/fba.2018-00067 (PMC6687334; doi:10.1096/fba.2018-00067)

Supplementary Figure Legends

Supplementary Figure 1. Morphology and characterization of *Vdr*-/- retinal pericytes (PC).

Morphology (A) and expression of known pericytes markers PDGF-Rβ, NG2, and SMA are shown (B). The representative mean fluorescence intensities are indicated in each histogram. Similar results were noted with two other isolations of these cells. Scale bar = 250 μm.

Supplementary Figure 2. The majority of VEGF produced by PC incubated with 1,25(OH)$_2$D$_3$ (Calcitriol) is in the secreted form.

The levels VEGF produced by PC incubated with calcitriol was determined by an ELISA using conditioned medium or cell lysates. The ELISA analysis confirmed that the majority of the detected VEGF is in the secreted form (conditioned medium) compared to the cell bound forms (cell lysates) in PC incubated with calcitriol (n = 3; ****P<0.0001, **P<0.01).

Supplementary Figure 3. Increased Vdr expression in retinal PC incubated with 1,25(OH)$_2$D$_3$ (Calcitriol).

The *Vdr* mRNA expression was assessed by qPCR analysis. Please note a significant increase in the VEGF mRNA detected in RNA prepared from PC incubated with calcitriol compared to vehicle control (n = 3; ****P<0.0001).

Supplementary Figure 4. Expression of VEGF receptors in *Vdr-/-* retinal PC.

VEGF-R1 and VEGF-R2 expression was assessed by FACS Scan analysis in *Vdr*-/-PC. The mean fluorescence intensities are indicted in the histograms. Similar results were observed in different isolations of these cells.

Supplementary Figure 5. Incubation with calcitriol did not dramatically affect the *Vdr-/-* retinal PC adhesion.

Although *Vdr*-/- PC were more adherent to the ECM proteins including collagen I, fibronectin, vitronectin, and collagen IV compared with *Vdr*+/+ PC, their adhesion properties remained unchanged in response to calcitriol. These experiments were repeated with two different isolation of these cells with similar results.

Supplementary Figure 1

A.

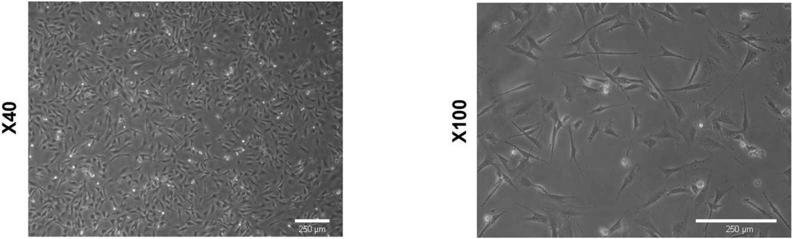

B.

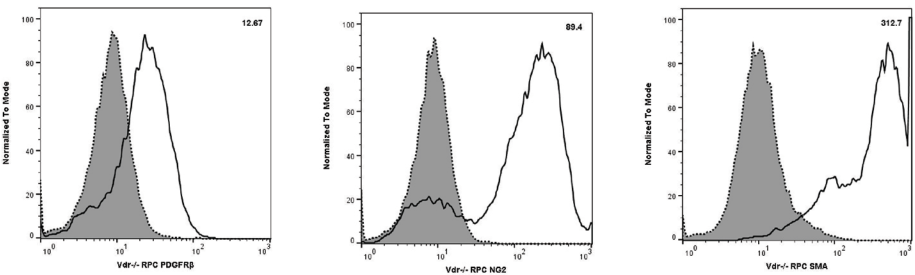

Supplementary Figure 2

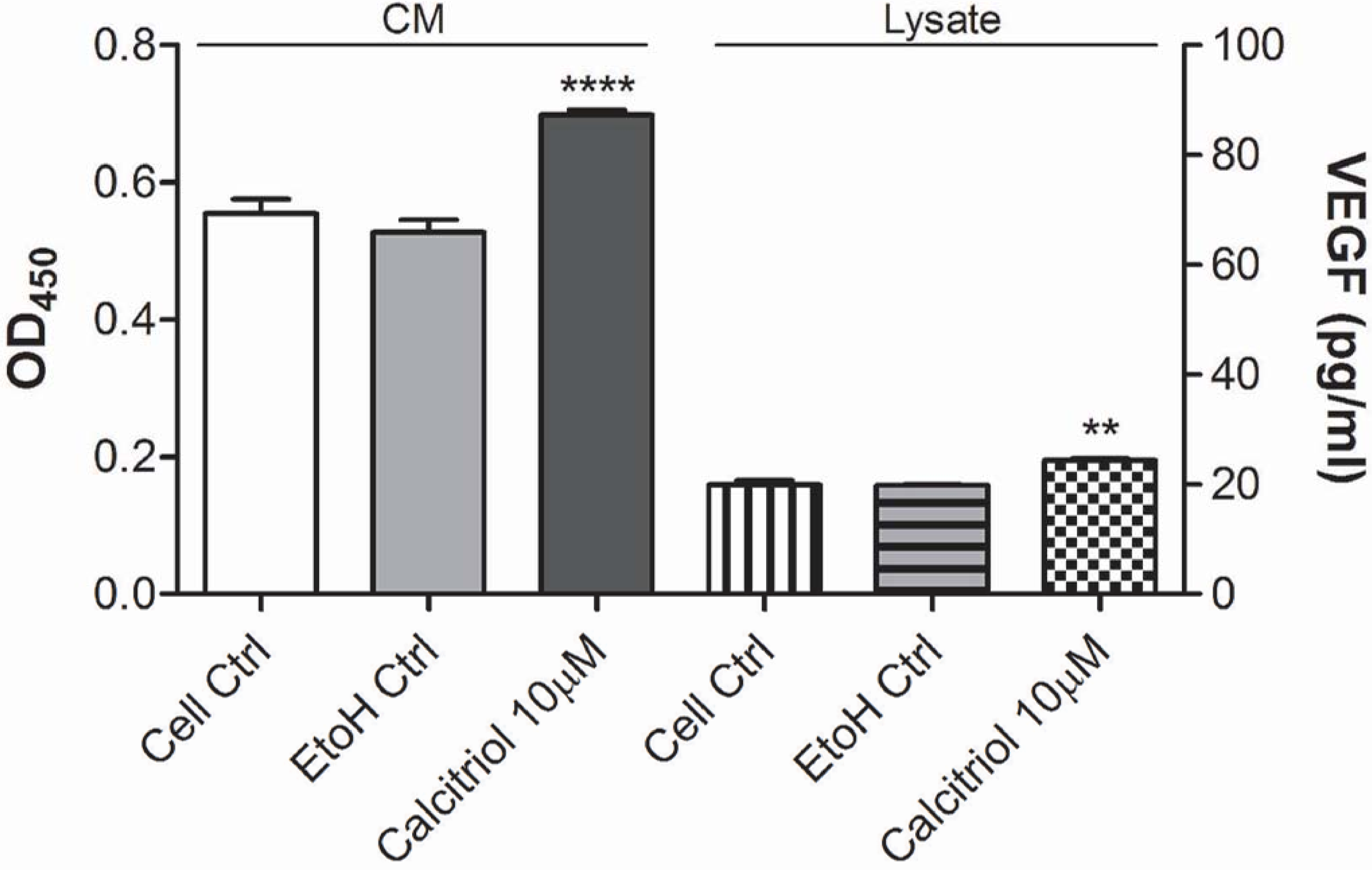

Supplementary Figure 3

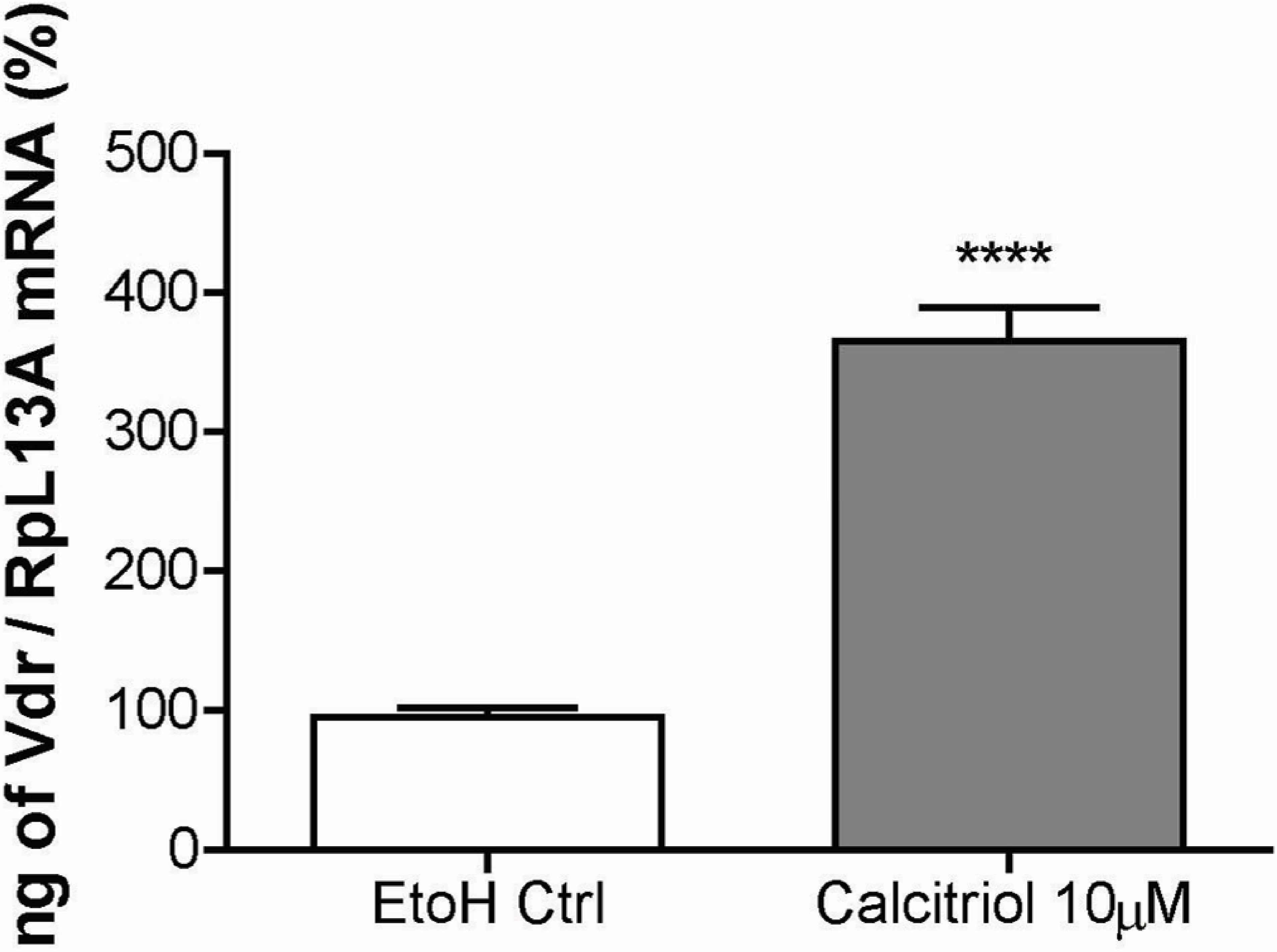

Supplementary Figure 4

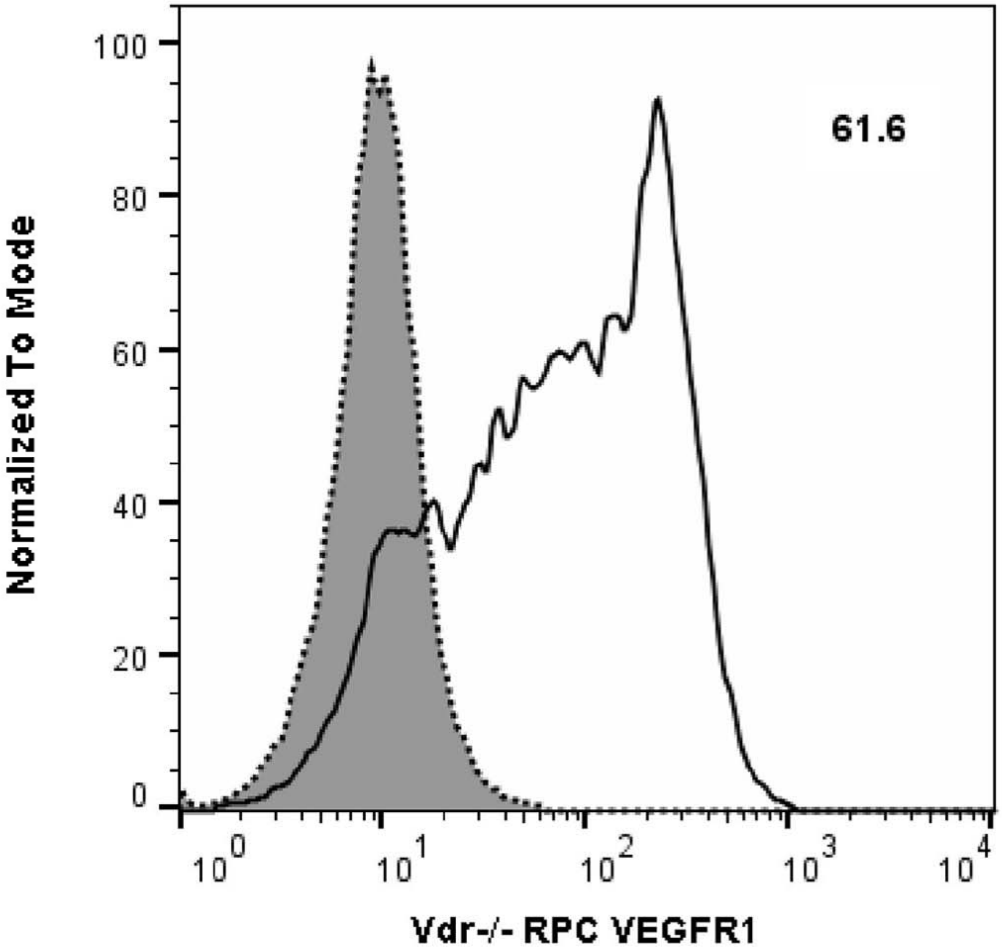

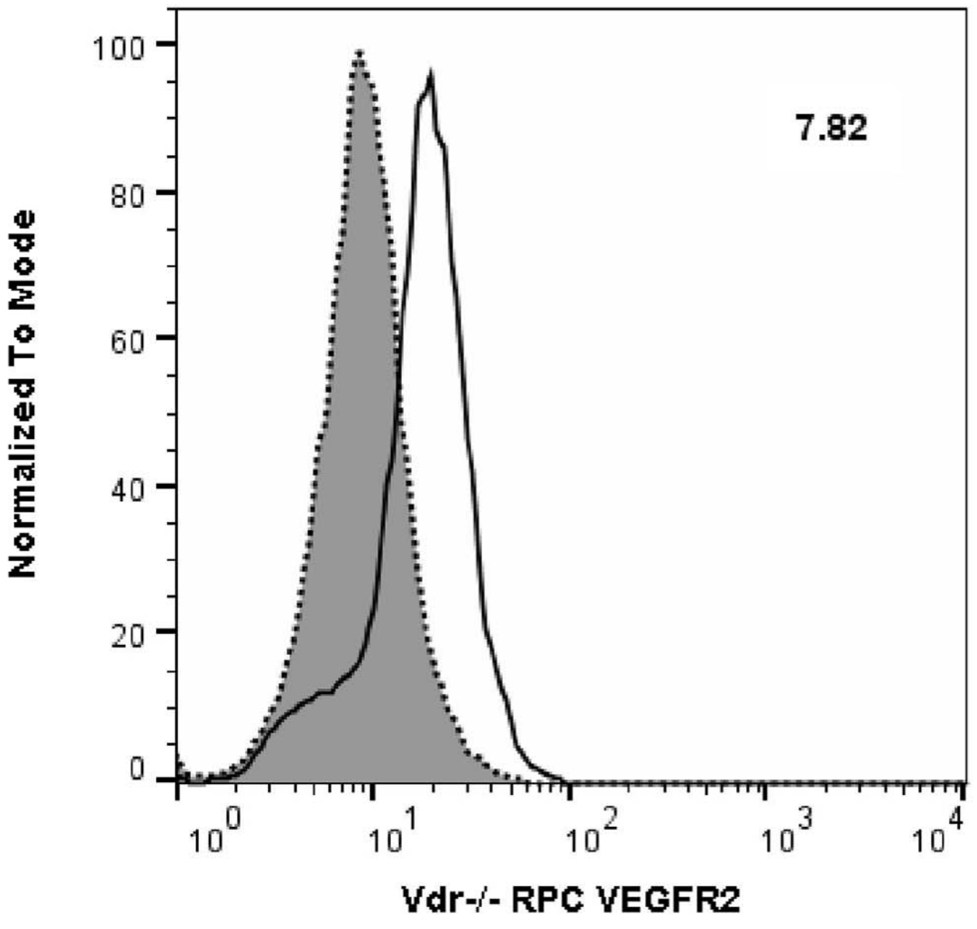

Supplementary Figure 5

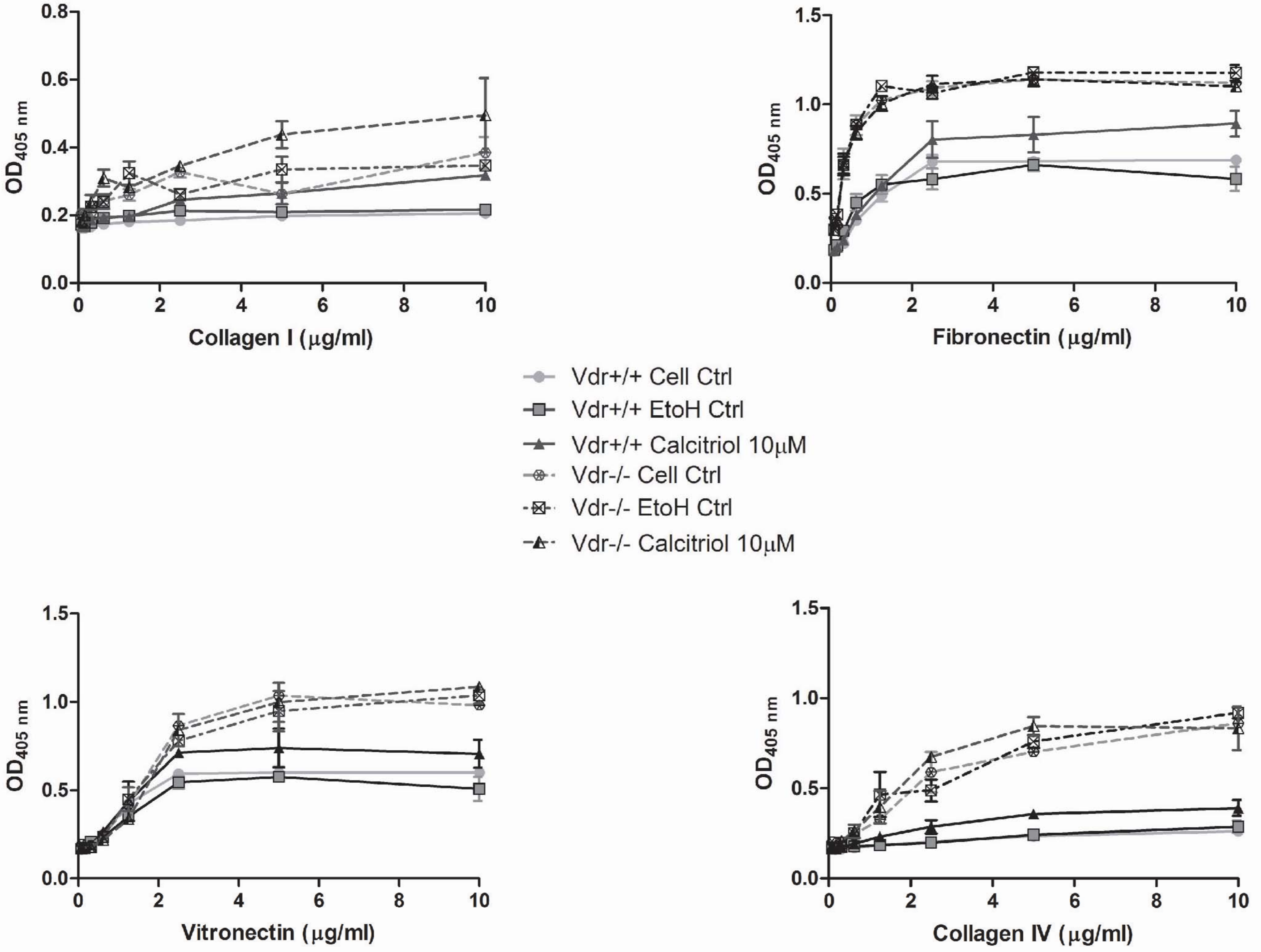

Supplement: Supplementary file 1 [file FBA2-1-415-s001.pdf]
